# Transcriptional Protein Sp1 Regulates LEDGF Transcription by Directly Interacting with Its *Cis*-Elements in GC-Rich Region of TATA-Less Gene Promoter

**DOI:** 10.1371/journal.pone.0037012

**Published:** 2012-05-16

**Authors:** Dhirendra P. Singh, Biju Bhargavan, Bhavana Chhunchha, Eri Kubo, Anil Kumar, Nigar Fatma

**Affiliations:** 1 Department of Ophthalmology and Visual Sciences, University of Nebraska Medical Center, Omaha, Nebraska, United States of America; 2 Department of Ophthalmology, Kanazawa Medical University, Ishikawa, Japan; 3 Department of Pharmacology, University of Missouri-Kansas City, Kansas City, Missouri, United States of America; Chinese University of Hong Kong, Hong Kong

## Abstract

LEDGF/p75 interacts with DNA/protein to regulate gene expression and function. Despite the recognized diversity of function of LEDGF/p75, knowledge of its transregulation is in its infancy. Here we report that *LEDGF/p75* gene is TATA-less, contains GC-rich *cis* elements and is transcriptionally regulated by Sp1 involving small ubiquitin-like modifier (Sumo1). Using different cell lines, we showed that Sp1 overexpression increased the level of LEDGF/p75 protein and mRNA expression in a concentration-dependent fashion. In contrast, RNA interference depletion of intrinsic Sp1 or treatment with artemisinin, a Sp1 inhibitor, reduced expression of LEDGF/p75, suggesting Sp1-mediated regulation of LEDGF/p75. *In silico* analysis disclosed three evolutionarily conserved, putative Sp1 sites within *LEDGF/p75* proximal promoter (−170/+1 nt). DNA-binding and transactivation assays using deletion and point mutation constructs of *LEDGF/p75* promoter-CAT revealed that all Sp1 sites (−50/−43, −109/−102 and −146/−139) differentially regulate *LEDGF/p75*. Cotransfection studies with Sp1 in *Drosophila* cells that were Sp1-deficient, showed increased LEDGF/p75 transcription, while in lens epithelial cells (LECs) promoter activity was inhibited by artemisinin. These events were correlated with levels of endogenous Sp1-dependent LEDGF/p75 expression, and higher resistance to UVB-induced cell death. ChIP and transactivation assays showed that Sumoylation of Sp1 repressed its transcriptional activity as evidenced through its reduced binding to GC-box and reduced ability to activate *LEDGF/p75* transcription. As whole, results revealed the importance of Sp1 in regulating expression of *LEDGF/p75* gene and add to our knowledge of the factors that control LEDGF/p75 within cellular microenvironments, potentially providing a foundation for LEDGF/p75 expression-based transcription therapy.

## Introduction

Gene expression is transcriptionally regulated through the interaction between *trans-acting* factors and the *cis*-elements of gene-specific promoters. A nuclear protein discovered relatively recently, LEDGF/p75 affects a variety of functions involved with cellular survival and development of cellular abnormalities [1], [2], [3], [4]. The functions of LEDGF/p75 protein are related to its expression level. Studies in cultured cell lines as well as *in vivo* have shown that overexpression of LEDGF/p75 gives growing cells a selective survival advantage by blocking death pathways [5], [6], [7]. LEDGF/p75 provides cytoprotection by acting as a transregulator of stress-associated genes such as Hsp27, -25, and -90, and αB-crystallin [8]. However, overexpression of LEDGF/p75 has been shown to be involved in subcutaneous angiogenesis and lymphangiogenesis of ovarian carcinoma tumors [9], and aberrant expression of LEDGF/p75 has been reported in 61% of prostate tumors [10]. These studies suggest that the level of LEDGF/p75 expression can determine the fate of cells in various cellular microenvironments.

LEDGF/p75 was originally identified as a transcriptional co-activator and transactivator that localizes predominantly in nucleus and binds to chromatin [11]. It performs various functions by interacting with protein/DNA [12]. LEDGF/p75 shares the first 325 amino acids with p52, an alternative splice variant from the same gene, mapping to chromosome 9p22.3 genetic locus [11], [13]. LEDGF/p75 is a multi-domain flexible nuclear protein. Reports indicate that PWWP and A/T hook domains of N-terminal LEDGF/p75 are involved in tethering of the lentiviral preintegration complex and chromatin [12]. C-terminal of LEDGF/p75 containing the integrase binding domain (IBD; residues 347 to 429) [14] binds to integrase and facilitates HIV integration with chromosome. Recently LEDGF/p75 has been shown to interact with multiple proteins such as Myc-interacting protein JPO2 [14] and mixed-lineage leukemia (MLL)/menin complex [15], a domesticated transposase PogZ (pogo transposable element derived protein with zinc finger) [14], Cdc7-activator of S-phase kinase (ASK) [16], and methyl CpG Binding Protein MeCP2 [17]. LEDGF/p75 protein has been shown to be a substrate of Sumo (small ubiquitin-like modifier), and Sumoylation of LEDGF/p75 negatively regulates its half-life and transcriptional activity [18]. Moreover, its helix-turn-helix (HTH)-like motifs (amino acids 421–442 and amino acids 471–492) bind to heat shock element (HSE, nGAAn) and regulate transcription of small heat shock protein genes (hsps). N-terminal LEDGF/p75 has been found to interact with stress-related response elements (STRE, nA/TGGGGA/Tn), thereby regulating transcription [2], [5], [8], [19], [20] and enhancing cell survival. Most importantly, LEDGF/p75 binding is not restricted to HSE or STRE, but LEDGF/p75 also binds to markers of active chromatin as well as RNA polymerase II, and correlates with transcriptional activity of the transcriptional unit [21]. More recently, LEDGF/p75 was found to selectively bind to supercoiled DNA and to recruit its binding partners to active transcription units [22]. Taken together, these reports indicate wide-spectrum activity of LEDGF/p75, and underscore its biological importance. The DNA-binding activity of LEDGF/p75 appears to be attributable to cellular microenvironment and cell background. During stress, LEDGF/p75 binds to stress response element(s), while under normal physiological conditions it interacts with chromatin/DNA.

Sp1, a prototypic C2H2-type zinc finger containing DNA binding protein, can transactivate or repress transcription in response to physiologic or pathologic stimuli. Recently, Sp1 was shown to be a regulator of several genes implicated in controlling many cellular phenomena including growth, [23], differentiation [24], apoptosis [24], angiogenesis [24] and immune response [24]. The multicellular functions of Sp1 involve the action through which it regulates gene transcription. Sp1 binds to GC-rich Sp1-responsive element (GC-box) with greater affinity [24] and can regulate TATA-less or TATA-containing gene promoter by direct binding to GC box or protein-protein interactions or by recruiting cofactors and other transcription factors. Many gene promoters are known to be regulated by Sp1 [25], [26], [27], although studies of model genes have revealed diverse mechanisms by which inducible transcription can be regulated. In fact, Sp1 was originally known as a constitutive activator of housekeeping genes, and recent reports indicate that posttranslational modification of Sp1 regulates its transcriptional activity and integrity [28]. Studies have shown that Sp1 undergoes Sumo1 conjugation [29], and that Sumoylation of Sp1 reduces its transcriptional potential [29]. Sumoylation/desumoylation is a dynamic process that maintains cellular signaling by conjugating or deconjugating Sumo1 to protein substrate(s). Like DUBs (deubiquitinases) opposing ubiquitination, members of the Sentrin/Sumo-specific proteases (Senp) enzyme family remove Sumo conjugated substrate to control protein function [30]. However, the potential effect of the Sp1 Sumoylation and desumoylation process on regulation of *LEDGF/p75* transcription remains unknown. It is also unclear whether Sp1 regulation influences LEDGF/p75 downstream target genes. In previous studies, we found that *LEDGF/p75* gene promoter ranging from −315 to +35 was sufficient for *LEDGF/p75* promoter activity [20]. In the current study, a careful analysis of the *LEDGF/p75* promoter using bioinformatics tools showed that the gene promoter was TATA-less and highly GC-rich and contains three putative Sp1-responsive elements. Several other regulatory elements predicted were heat shock and stress-response elements, VDR/RXR (vitamin D receptor/retinoid X receptor), STAT, E2F, OCT1, GRE, Sp1, GATA-1, IRF-1 and IRF-2, including the TIE sites at −444 to −433 from the transcription start site [20]. Furthermore, an extensive literature survey revealed that Sp1 is largely associated with regulating TATA-less promoter [31], and may be a transregulator of the *LEDGF/p75* gene promoter that lacks the canonical TATA box consensus transcription. From the dynamic systems point of view, we also studied how LEDGF/p75 expression is fine tuned by the regulatory mechanism Sumoylation of Sp1, and how, at a critical level, Sp1 activity is reversibly engaged in favor of cellular integrity.

In this work, we report that Sp1 transactivates the human *LEDGF/p75* gene; in fact, the finding that Sp1 is a ubiquitous transcriptional protein is consistent with the expression of *LEDGF/p75* gene in cells. We show that the 5′-flanking region sequences of the human *LEDGF/p75* gene are devoid of CCAAT and TATA-boxes, that is a TATA-less promoter and that the minimal promoter is enriched with GC content. We have determined the Transcription start site (TSS) and characterized GC-rich DNA sequence motif in the promoter responsible for regulation of *LEDGF/p75* transcription. GC-rich transcriptional control elements are always complicated by the relatively large number of DNA-binding proteins that are capable of interaction with GC-rich sequences [32]. However, we were able to identify three Sp1-responsive elements in the proximal region of *LEDGF/p75* promoter and determine the function and contribution of each Sp1 regulatory element in *LEDGF/p75* gene transcription. Also, we provide evidence that *LEDGF/p75* transcription is controlled by Sp1 posttranslational modification, Sumoylation/desumoylation. We found that Sp1 overexpression increased LEDGF/p75 mRNA and protein expression in cells, and these cells gained resistance against UVB stress. Our studies revealed, for the first time, the mechanism by which Sp1 regulates LEDGF/p75 expression. This regulation may be attributed to cell survival response, by avoiding any aberrant expression of LEDGF/p75 that would cause cellular abnormalities.

## Results

### Sp1 expression-dependent abundance of LEDGF/p75 mRNA and protein in hLECs demonstrated that Sp1 may be a regulator of LEDGF/p75

**Figure 1:**
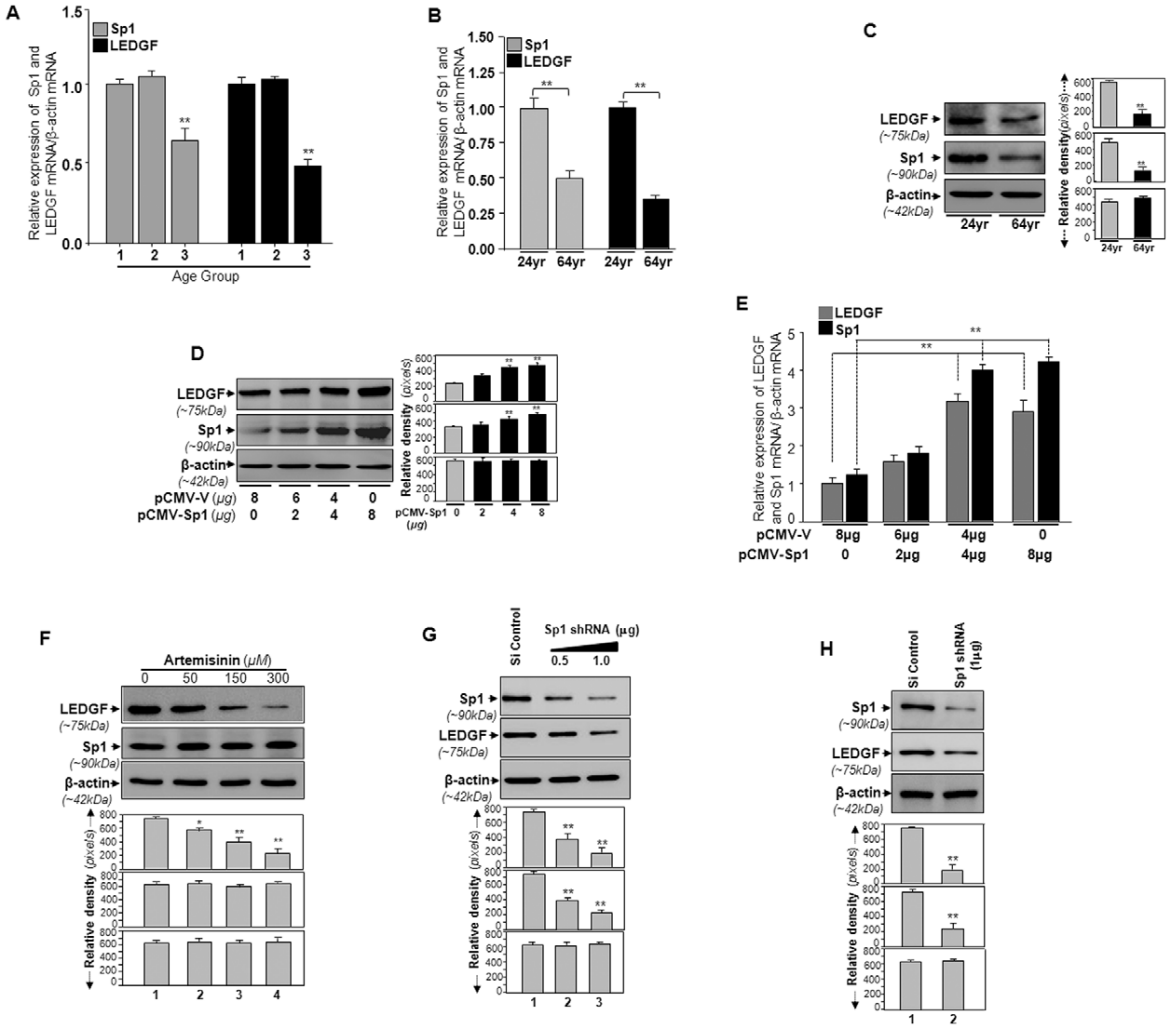
Expression pattern of LEDGF/p75 in LECs from human eye lenses of different ages was associated with Sp1 expression. A and B, mRNA expression levels of LEDGF/p75 (*black bars*) and Sp1 (*gray bars*) were analyzed by real time PCR. Total RNA was isolated from LECs separated from lenses of human subjects of different age groups and reverse transcribed cDNA was subjected to real time PCR analysis with specific primers as detailed in Materials and Methods. Age group 1 (n = 4, 16–26 years); Age group 2 (n = 3, 34–42 years); Age group 3 (n = 7, 52–75 years). n; denotes number of subjects. The data represent the mean ± S.D. from three independent experiments (** *p<0.001*). C, Western analysis of LEDGF/p75 and Sp1 protein using their corresponding specific antibodies. hLECs isolated from eye lenses of 24- and 64-year-old human subjects were cultured as described in Materials and Methods. Cellular proteins from confluent cells were extracted, and equivalent amounts were loaded onto SDS-PAGE, transferred to a PVDF membrane and processed for immunoblotting. Western analysis showed the expression levels of LEDGF/p75 (*upper* panel) and Sp1 (*middle* panel). *Lower* panel, membrane probed with β-actin antibody as loading/internal control. The same membrane was probed and reprobed with antibodies following stripping and restriping to obtain relative expression of Sp1, LEDGF/p75 or β-actin. Each band of blot was quantified using densitometer shown at the *right*. Images are representatives from three independent experiments. D and E, Sp1 upregulated expression of LEDGF/p75 protein and mRNA in hLECs in dose dependent fashion. hLECs were transfected with either pCMV-vector or increasing amounts of pCMV-Sp1 (2, 4 and 8 µg) as indicated and described in Materials and Methods section. Total Protein and RNA were extracted after 48 h of transfection and were used for Western analysis (D) and real time PCR (E) respectively, using specific probes. D, *left*, Western analysis data showing the expression levels of LEDGF/p75 (*upper* panel) in cells transfected with plasmid encoding Sp1 at different concentrations (*middle* panel). *Lower* panel, membrane probed with β-actin antibody. The same membrane was probed and reprobed with antibodies following stripping and restriping to obtain relative expression of Sp1, LEDGF/p75 or β-actin. *Right*, Histogram displaying relative protein band density indicated as values ± S.D. of three independent experiments. E, Histogram showing the values (mean ± S.D.) of Sp1 concentration-dependent expression of LEDGF/p75 mRNA (black bars *vs* gray bars) obtained from three independent experiments (**p<0.001). F, A Sp1 inhibitor, artemisinin, reduced expression of LEDGF/p75 in LECs in dose-dependent manner. Cultured cells were treated with either increasing concentrations of artemisinin (50, 150 and 300 µM) or with vehicle control. Cell lysates were resolved onto SDS-PAGE and analyzed by Western blot for the effects of artemisinin on expression of LEDGF/p75 and Sp1 protein. Relative band density in pixels is shown *below* the Western blot images (*p<0.01, **p<.001). β-actin was used as internal control. G and H, Representative immunoblots showing depletion of Sp1 using Sp1 Knockdown assay. Sp1-specific shRNA constructs were transiently (G) and stably (H) transfected as described in Materials and Methods section. Protein lysate was prepared and Western analysis was carried out. The same membrane was probed and reprobed with antibodies following stripping and restriping to obtain relative expression of Sp1 or LEDGF/p75 or β-actin. Relative band density in pixels is shown below the Western blot images (**p<.001).

Both LEDGF/p75 and Sp1 are ubiquitously expressed and play roles in controlling cellular survival, differentiation, and proliferation [3], [4], [24]. Their aberrant expression alters normal cellular signaling, leading to cell abnormalities such as cancer and its progression [3], [10], [15], [24]. We envisaged that Sp1 might be a regulator of LEDGF/p75 expression. We examined the relative expression pattern of LEDGF/p75 and Sp1 protein and mRNA in hLECs derived from eye lenses of subjects aged 16–75 years, and divided them into three groups: (group 1, 16–26 y; group 2, 34–42 y, and group 3, 52–75 y (Fig. 1A). Results revealed that the changes in expression pattern of both molecules were sequentially similar in all age groups tested. As shown in Fig. 1A, expression levels of LEDGF/p75 mRNA (black bars) and Sp1 mRNA (gray bars) were well correlated. We also examined protein level and mRNA levels in LECs isolated from 24- and 64-year-old subjects, and found that expression patterns of Sp1 mRNA (Fig. 1B) and protein (Fig. 1C) directly correlated with expression levels of LEDGF/p75 protein and mRNA. These data indicated that changes in LEDGF/p75 expression pattern can be associated with changes in Sp1 expression.

Next, to test whether LEDGF/p75 expression is indeed induced by Sp1, we ectopically expressed human Sp1 by transfecting cells with different concentrations of pCMV-Sp1 (0, 2, 4, 8 µg) or pCMV-empty vector as described in the Materials and Methods section. The extracted protein from transfectants was resolved by SDS-gel electrophoresis and blotted onto nitrocellulose membrane. To ascertain equal loading and relative expression levels of LEDGF/p75, Sp1 and β-actin (internal control), we continued with the same blotted membrane to probe or reprobe after stripping with antibody specific to LEDGF/p75 or Sp1 or β-actin. As predicted, cells overexpressing Sp1 displayed elevated expression of LEDGF/p75 protein (Fig. 1D, *upper* image), and expression of LEDGF/p75 was dependent on Sp1 concentration (Fig. 1D, *middle* image). The same blotted membrane immunostained with β-actin antibody did not reveal any altered expression in β-actin (Fig. 1D, *lower* image), suggesting that LEDGF/p75 protein was selectively and specifically increased by Sp1. We next examined whether cells with higher levels of Sp1 displayed higher LEDGF/p75 mRNA. As described above, Sp1 transfectants containing different concentrations of Sp1 plasmid were harvested. RNA isolated from these cells was processed for real-time PCR. The expression levels of LEDGF/p75 mRNA were significantly increased in cells overexpressing Sp1, and the increases were dependent on Sp1 expression (Fig. 1E, black bars *vs* gray bars). Collectively, these observations revealed that Sp1 overexpression enhanced the expression of LEDGF/p75 mRNA in hLECs, and we found a significant direct correlation between Sp1 and LEDGF/p75 expression.

### Artemisinin, an inhibitor of Sp1, or SiRNA knockdown of SP1 downregulated expression of LEDGF/p75 protein

As a further step toward understanding whether Sp1 is involved in increased expression of LEDGF/p75, we treated LECs with various doses of artemisinin (ART). Previous studies established that ART inhibits regulatory activity of Sp1 to its target gene expression [33]. LECs cultured with variable concentrations of ART (0, 50, 150, 300 µM) were processed for Western analysis. Anti-LEDGF/p75 immunostaining revealed a concentration-dependent decrease in LEDGF/p75 protein expression in treated LECs (Fig. 1F), while no change occurred in expression level of β-actin (internal control), demonstrating that ART specifically and selectively inhibited Sp1-dependent expression of LEDGF/p75. ART did not affect the expression level of Sp1 (Fig. 1F, middle panel), indicating that transcriptional activity of Sp1 was essential for LEDGF/p75 expression.

To further confirm direct involvement of Sp1 in activating LEDGF/p75 transcription, we depleted endogenous Sp1 expression either by transiently (Fig. 1G) or stably (Fig. 1H) transfecting cells with shRNA specific to Sp1 as described in the Materials and Methods section. The expression level of endogenous Sp1 was markedly and specifically reduced by transfection of Sp1 shRNA in concentration-dependent fashion, but Si control did not show altered expression level of Sp1. Next we analyzed expression level of LEDGF/p75 by reprobing the same membrane with antibody specific to LEDGF/p75. As expected, the expression level of endogenous LEDGF/p75 was decreased, and the decrease was directly correlated with expression levels of Sp1. In contrast, Si control did not alter the level of LEDGF/p75 (Fig. 1G and 1H). Collectively, the data indicate that Sp1 regulates LEDGF/p75 expression.

### Bioinformatic analysis of 5′-flanking sequence showed that human *LEDGF/p75* gene is a GC rich TATA-less promoter containing three putative Sp1 regulatory elements

**Figure 2:**
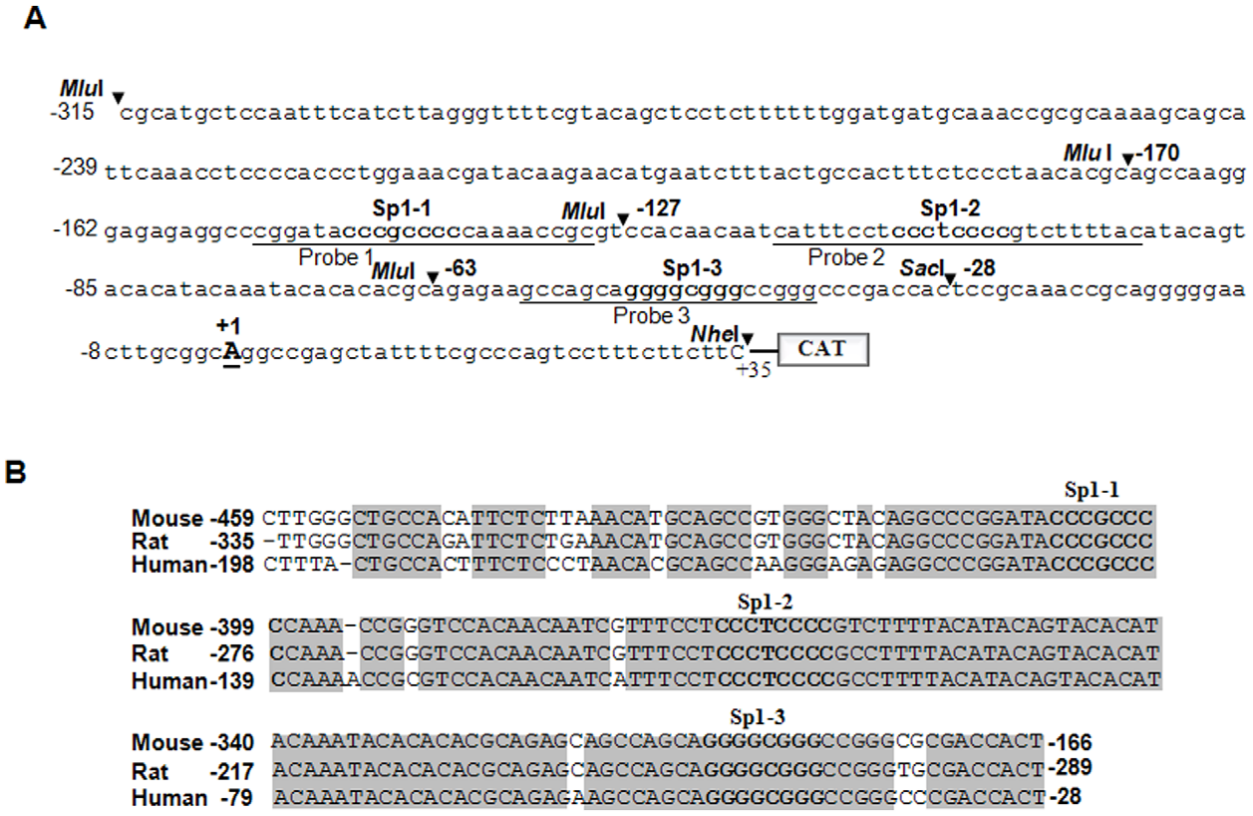
A construct linking the 5′ proximal promoter region of the TATA-less *LEDGF/p75* promoter to CAT reporter gene showing putative characteristic features. A, The 5′- sequence ranging from nucleotides −315 to +35 contained three putative Sp1 binding sites as predicted by MatInspector (Genomatix). The consensus sequences for the predicted Sp1 sites (G/C boxes) are shown in **bold** and sites are denoted as Sp1-1, Sp1-2 and Sp1-3. Underlining is used to show the oligonucleotides employed in gel-shift and gel-shift immuno-deletion assays. The transcription start site is indicated by **+1**, and letter *Mlu* I and *Nhe* I restriction sites used for preparing *LEDGF/p75*-CAT constructs are shown in italic and bold. B, Nucleotide sequences alignment of the proximal promoter of mouse, rat and human *LEDGF/p75* gene (NCBI, BLAST and alignment tools). Sequences highlighted in gray are highly conserved among these species, and three evolutionarily conserved Sp1 binding sites are shown in bold letters.

In previous reports, we described the structural organization of *LEDGF/p75* protein [34] and cloning of genomic fragments containing the 5′-flanking region of *LEDGF/p75* ranging from −5139/+35, and we identified the proximal promoter essential for *LEDGF/p75* promoter activity [20]. Since the patterns of core promoter may differ in different cells due to variability in transcription start site (TSS), we determined TSS of *LEDGF/p75* gene (S1) [35]. Using RNA isolated from hLECs, we found that the *LEDGF/p75* gene contained three TSSs; one major and two minor, as shown in Fig. S1. We identified the location of core promoter of LEDGF/p75 based on major TSS. In the current work we attempted to delineate the regulatory element(s) responsible for *LEDGF/p75* gene transcription. Sequence analysis of *LEDGF/p75* gene showed that the proximal region (−170/+35) was relatively enriched with G/C content and, most importantly, had an apparent absence of CCAAT and TATA boxes (Fig. 2A) as is common with many GC-rich promoters. In TATA–less promoter, Sp1 regulatory element appeared to play a pivotal role in gene transcription [36]. Further analysis of the promoter region using MatInspector (Genomatix) revealed the presence of three Sp1-like binding sites within the G/C rich region (Fig. 2A and 2B, Sp1-1(nCCCGCCCCn), Sp1-2 (nCCCTCCCCn), and Sp1-3 (nGGGGCGGGn). The sites consisted of heterogeneous sequences that matched all or at least five of the six nucleotides for a canonical Sp1 binding site [24]. However, consensus sequences for predicted Sp1-1 and Sp1-2 binding sites were on antisense strand, and Sp1-3 was on the sense-strand of *LEDGF/p75* gene. Sp1 sites present in either orientation have been shown to be capable of activating transcription [24], [36]. Interestingly, Sp1 sequences in *LEDGF/p75* gene can be classified into two parts, a GC rich region containing consensus Sp1 sequence (Sp1-3), and a region enriched with T/TCCCC repeats bearing Sp1-1 and Sp1-2 sites. Furthermore, a comparison of *LEDGF/p75* 5′-flanking region sequences among mouse, rat and human cells revealed that proximal region containing Sp1 regulatory elements is highly conserved among them (Fig. 2B), indicating the importance of Sp1 regulatory elements in *LEDGF/p75* promoter.

To identify and characterize the functionality of Sp1 sites and their contribution to regulating *LEDGF/p75* transcription, we conducted transfection and transactivation experiments, and correlated results with the endogenous expression pattern of LEDGF/p75. We also utilized Sp1-deficient *Drosophila* cell lines (SL2).

### Transcriptional analysis revealed three functional Sp1 regulatory elements of human *LEDGF/p75* promoter

**Figure 3:**
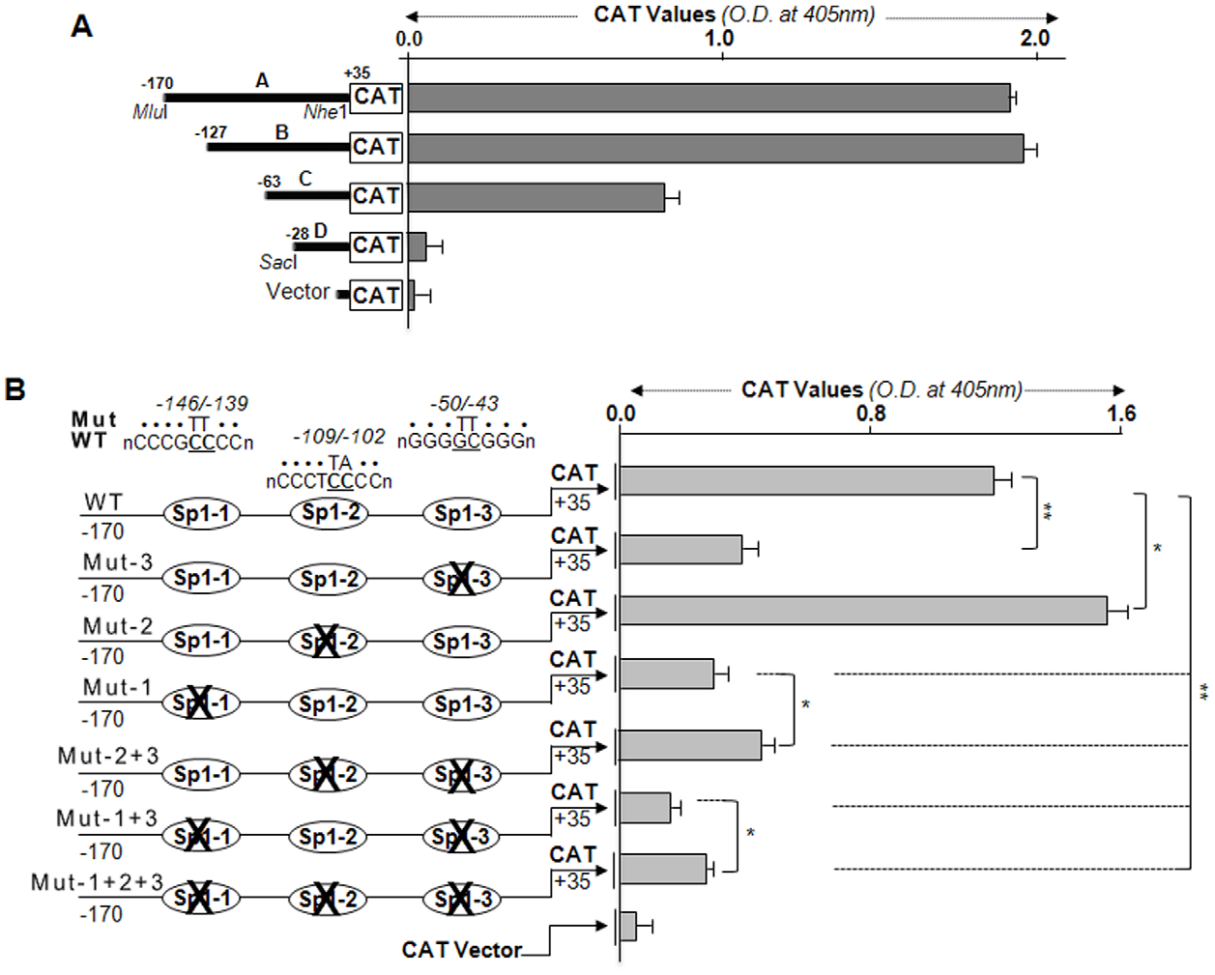
Transcriptional activity of *LEDGF/p75* promoter and identification of functioning potential of Sp1 sites within human *LEDGF/p75* promoter. A, *Left* half, diagrams showing the 5′-deletion constructs of *LEDGF/p75* promoter linked to CAT reporter gene used for transient transfections. *Right* half, CAT activity of the *LEDGF/p75* promoter deletion constructs and empty CAT vector in hLECs. 5′-deletion mutant constructs and pGFP were cotransfected into hLECs. 48 h later, protein was extracted and CAT activity was measured. CAT activity (*right*) was normalized to GFP readings (O.D.). The data represent the mean ± S.D. from three independent experiments. B, Point mutation analysis showing Sp1 site-dependent transcriptional activity of the *LEDGF/p75* gene promoter in hLECs. *Left* half, schematic representation of Sp1-site-directed mutants of *LEDGF/p75* promoter linked to CAT. *Right* half, CAT activity of the wild-type (WT) and its mutant constructs (Mut-3, Mut-2, Mut-1 and Mut- 1+2+3) and empty CAT vector in hLECs. All data are presented as the mean ± S.D. derived from three independent experiments (*p<0.01, **p<0.001).

In a previous report [20], using a series of mutation deletion constructs of *LEDGF/p75* promoter linked to TSS (Fig. 2A, **+**1). However, regulatory elements involved in *LEDGF/p75* transcription were not characterized. In the present study, we engineered a series of deletion mutant constructs linked to CAT (Fig. 2A) to further define functioning of promoter region containing Sp1 sites with common 3′ end (+35). In the transactivation assay (Fig. 3A), construct (−170/+35) containing three Sp1 putative sites or construct (−127/+35) with two showed transcriptional activity, while in Construct C, containing only one Sp1 site (−63/+35), promoter activity was significantly reduced. However, in Construct D, with no Sp1 site, the CAT activity was insignificant and was comparable to CAT vector activity alone. Data analysis demonstrated that the functional *cis* regulatory elements may reside in the approximately 144 bp region between −170 and −28.

Next, we examined the activity of each Sp1 site predicted by Web-based analysis (MatInspector, Genomatix). Data from transactivation experiments with different deletion mutant constructs of *LEDGF/p75* promoter implied that the positive regulatory element(s) lay in the proximal promoter region spanning from −170 to −28 bps (Fig. 3A). This region contained all three Sp1 sites, suggesting that all may contribute cooperatively and efficiently to regulate *LEDGF/p75* gene transcription. To test the functionality and contribution of each Sp1 site present in the region, we made a series of point mutations in core consensus sequences that disrupted the Sp1-binding sites (Fig. 3B). In Sp1-Mut-3 (−50/−43) site, **GC** to **TT**; Sp1-Mut-2 (−109/−102) site, **CC** to **TA**; and Sp1-Mut-1 (−146/−139) site, **CC** to **TT** were mutated. Sp1 is known to play a role in the regulation of GC-rich genes lacking a TATA box. Transactivation assay with mutant promoters in LECs revealed that disruption of any of Sp1-Mut-3 and Sp1-Mut-1 resulted in significantly reduced promoter activity (p<0.001). Surprisingly, mutant construct Sp1-Mut-2 released the promoter activity dramatically in contrast to the other two Sp1 sites (Fig. 3B), showing this site acted as a repressor. Mutation of the Sp1-Mut-3 or Mut-1 site showed greater reduction in promoter activity, and those sites had similar activation potential, demonstrating that both had acted as transactivator [Fig. 3B, Sp1-3 (Mut-3) and Sp1-1(Mut-1)].

We also examined combinatorial effects of Sp1 sites. Sp1-Mut-2+3 with double mutation displayed promoter activity similar to Sp1-Mut-3 and Sp1-Mut-1, suggesting that the repressive function of Sp1-Mut-2 site was attenuated significantly in the presence of Sp1-3 as an activator. Double mutation at Sp1-Mut-1+3, however, showed further reduction in promoter activity, emphasizing that the nature of regulatory activity is the same for both sites. The construct Sp1-Mut-1+2+3, in which all three sites were disrupted, showed reduction in promoter activity similar to that with mutant construct (Sp1-Mut-1+3) (Fig. 3B), suggesting that all sites function in the control of *LEDGF/p75* promoter activity under normal cellular physiological conditions. Similar results were obtained in other cell lines (mouse lens epithelial cells [mLECs] and Cos7 cells, data not shown).

Notably, the promoter activity of mutated constructs (Sp1-Mut-2) did not attenuate WT-promoter activity, but rather released and enhanced the activity when compared to wild-type (Fig. 3B, Sp1-WT *vs* Sp1-Mut-2, p<0.01) or other mutant promoter, and the increase in activity was dependent on Sp1-Mut-2 site. We think that each of the three sites of Sp1 in *LEDGF/p75* gene promoter has different transactivation potential, and thus the sites responded differently. This suggests that Sp1 is a regulator and is also involved in controlling the magnitude of transcription required for cellular background. In addition, variation in consensus sequences in Sp1 sites may change binding affinity that influences the transactivation potential of Sp1 [32]. We believe that the diverse sequences from consensus binding sites (sequences) were evolved during natural selection for cellular benefit. Thus, each of the Sp1 sites contributes to controlling *LEDGF/p75* transcription. Two Sp1 responsive element sites, −50 to −43 and −146 to −139, are essential for activation of *LEDGF/p75* gene, while an Sp1 site at position −109 to −102 represses the promoter activity. Collectively, the data provide evidence that Sp1 regulatory elements in *LEDGF/p75* promoter are responsible for LEDGF/p75 expression.

### DNA-binding assay revealed that Sp1 in nuclear extracts of cells directly and selectively interacted with its regulatory elements in the *LEDGF/p75* promoter

**Figure 4:**
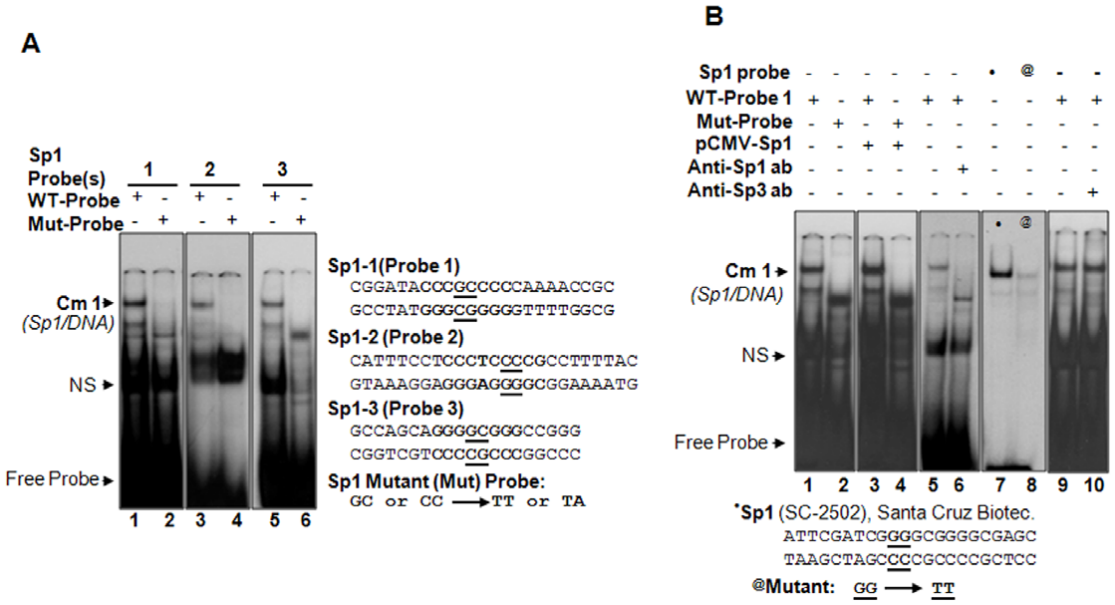
Nuclear extract from LECs bound to Sp1 sites present in human *LEDGF/p75* promoter. A, Representative gel-shift mobility assays showing Sp1 binding to radiolabeled oligonucleotide probes containing consensus Sp1 sites as indicated. Nuclear extracts isolated from hLECs were incubated with ^32^p-labeled probes containing Sp1 binding sites (WT-probes) or their corresponding mutants (Mut probes). Nuclear extracts bound to oligos containing Sp1 sites and yielded to complex, Sp1/DNA (Cm1) (A, lanes 1, 3 and 5). No complex occurred with mutant probes (A, lanes 2, 4, and 6). The oligonucleotide probes of both wild-type and mutated sequence used in assay are shown adjacent to image. B, Gel-shift assay showing the binding of Sp1 in nuclear extract of Sp1 overexpressed with hLECs to ^32^p-labeled probes with its site. Nuclear extract isolated from cells transfected with plasmid encoding Sp1 or its corresponding vector was incubated with WT-probe1 or standard control probe (sc-2502; Santa Cruz Biotech). The DNA-protein complex was resolved on a 5% acrylamide gel. A discrete Sp1 expression-dependent DNA-protein complex was observed (B; lanes 1 *vs* 3) in comparison to vector transfected cells (lane 1), while the mutated probe failed to generate the complex (B, lanes 2 and 4). B, *Right (lanes 5 and 6)*, depletion of endogenous Sp1 with its specific antibody. Nuclear extracts were incubated with either anti-Sp1 antibody (lane 6) or normal rabbit IgG (lane 5), and recovered nuclear extracts were incubated with the same probes (lanes 5 and 6). Lanes 7 and 8, standard control containing ^•^Sp1 site (sc-2502, Santa Cruz Biotech) or its ^@^mutant (sc-2503) processed for gel-shift assay using the same nuclear extracts. *Extreme right*, Depletion assay using anti-Sp3 antibody with nuclear extract showing no change in Sp1/DNA complex (lane 10) and the complex was indistinguishable from Lane 9. Images are representatives from three independent consistent observations.

To examine whether Sp1 regulatory elements (Fig. 2A, Sp1-1, Sp1-2 and Sp1-3) in the human *LEDGF/p75* promoter directly bound to Sp1 in nuclear extract of hLECs, double stranded DNA oligonucleotides containing Sp1 site(s) (as in *LEDGF/p75* promoter, Fig. 2A) and their mutants were chemically synthesized. These ^32^p-radiolabelled probes (Fig. 4) were utilized in gel-shift assay. All oligonucleotides showed sequence-specific binding of Sp1 proteins, although some sequences differed from the defined canonical Sp1 consensus sequence [37]. A representative gel-shift assay using all three WT-probes and their mutants is shown in Fig. 4A. All three DNA probes formed complex (Sp1/DNA) with nuclear extract (Fig. 4A, lanes; 1, 3 and 5). In contrast, their corresponding mutant probes did not form Sp1/DNA complex with the nuclear extract (Fig. 4A: lanes 2, 4 and 6) (Fig. 4A: Mutant probe; underlined nt, G to T/A and C to T/A). A band (NS) appeared in all lanes with approximately the same intensity, signifying a nonspecific entity.

Next, we examined whether the increased abundance of Sp1 protein in nuclear extract would show increased binding to oligo-containing Sp1 sites. We over expressed hLECs with Sp1 by transfecting them with pCMV-Sp1 eukaryotic expression constructs. Nuclear extract derived from these cells interacted with probe (WT-Probe1) and gave rise to Sp1/DNA complex (Cm1) of higher intensity than in the control, which did not overexpress Sp1 (Fig. 4B; lane 1 *vs* 3). No Sp1/DNA complex was detected with a mutant probe containing disrupted Sp1 consensus (Fig. 4B, lanes 2 and 4), further suggesting that Sp1 selectively bound to its sites. We also verified the integrity of DNA and protein (nuclear extract) interaction by using standard control probe containing Sp1 sites (sc-2502 or its mutant sc-2503) and Sp1 in nuclear extract. Fig. 4B, lanes 7 and 8 show the binding of Sp1 in nuclear extract to the probe, suggesting consistent interaction among probes (Sp1-1, Sp1-2 or Sp1-3) bearing putative Sp1 sites. To examine whether binding of Sp1 was specific to probes, we conducted antibody depletion assay using antibody specific to Sp1. Nuclear extract absorbed with anti-Sp1 antibody showed reduced or no binding to oligo probes containing Sp1 site (Fig. 4B; lane 5 *vs* 6) compared to nuclear extract absorbed with normal rabbit IgG (Fig. 4B, lane 5), demonstrating that Sp1 bound specifically to probe. Since Sp1 and Sp3 bound the same consensus sequence elements [24], to ensure that Sp1 bound specifically and exclusively to probe, we performed gel-shift assay with antibody-depletion experiments by incubating antibody specific to Sp3 with nuclear extract. We found that Sp3 antibody did not influence complex mobility and integrity on gel, demonstrating that Sp3 did not occupy the Sp1-binding elements (Fig. 4B, lanes 9 and 10). Similar results were obtained in experiments with other two probes containing Sp1 sites or its mutant site (data not shown).

**Figure 5:**
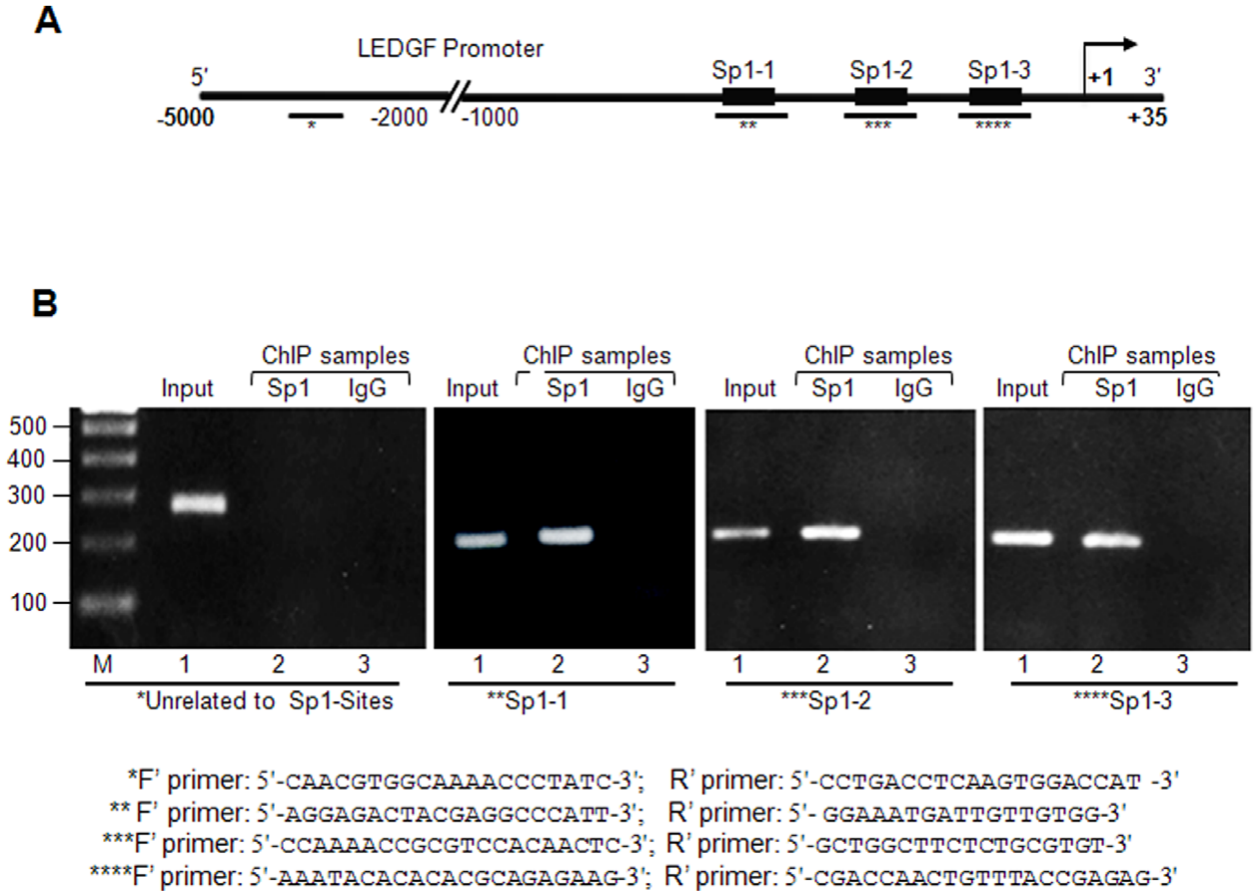
ChIP analysis of genomic DNA from hLECs revealed Sp1 binding to *LEDGF/p75* promoter *in vivo*. A, Schematic illustration of 5′-proximal promoter region of *LEDGF/p75* containing Sp1 binding sites. Genomic DNA was cross-linked to immobilize bound proteins *in vivo*, was sheared and immunoprecipitated with anti-Sp1 or unrelated antibody rabbit IgG, and was amplified by PCR with primer specific to the region. The quantity of each input DNA was initially measured equalized by O.D. A representative gel stained with ethidium bromide is shown. As a control measure, amplification of the −2499/−2277 region (*) devoid of Sp1 elements of *LEDGF/p75* promoter following immunoprecipitation was performed. B, Photographic image of the amplified DNA band visualized with ethidium bromide staining. M, molecular weight marker. *Lower* half, primers used for amplification of specific region containing Sp1 sites (^**^Sp1-1, ^***^Sp1-2, ^****^Sp1-3) and not related to Sp1 binding sites (^*^).

*In vitro* data suggested that Sp1 exclusively bound to its putative regulatory elements within the *LEDGF/p75* promoter. However, to determine if the regulation of *LEDGF/p75* transcription by Sp1 occurred via a direct mechanism *in vivo*, we employed chromatin immunoprecipitation assay (ChIP) to measure the occupancy of Sp1. We utilized identified Sp1 response element region in the *LEDGF/p75* promoter (Fig. 5A) and carried out PCR on chromatin DNA fragment specifically immunocomplexed to Sp1. After formaldehyde crosslinking and sonication to shear the chromatin, chromatin fragments were immunoprecipitated from cultured hLECs with the antibodies anti-Sp1 and control rabbit IgG. DNA from the immunoprecipitated complex was then recovered. From this DNA, a fragment of the *LEDGF/p75* promoter-containing Sp1 sites was amplified by PCR using a set of predesigned primers for the region (Fig. 5B). A pair of sense and antisense primers was also designed, along with a negative control to amplify a region of genomic DNA beyond 2 kb from Sp1 binding sites. As shown in Fig. 5B, Sp1 specifically bound to the *LEDGF/p75* promoter region containing the Sp1 sites (all three sites: −146/−139, −109/−102, −50/−43). No amplicons were examined with either primer for DNA beyond Sp1 sites or control IgG (Fig. 5B). These data demonstrate that Sp1 protein bound to the *LEDGF/p75* promoter, and protein DNA complex was immunoprecipitated by an anti-Sp1 antibody but not by irrelevant control IgG antibodies (Fig. 5 B, αIgG panels), pointing to the specificity of immunocomplex produced selectively by anti-Sp1 antibody. This assay did not reflect that all Sp1 sites are involved selectively or specifically, but gel-shift assay coupled with transactivation assay clearly indicated that all three Sp1 sites bound selectively and functionally to Sp1. The functional significance of this binding was further examined in transactivation assays using the Sp1 inhibitor artemisinin and/or cells overexpressing Sp1.

### Artemisinin and Sp1 transfection assays revealed that *LEDGF/p75* transactivation was largely derived from direct functional binding of Sp1 to *LEDGF/p75* promoter *in vivo*

**Figure 6:**
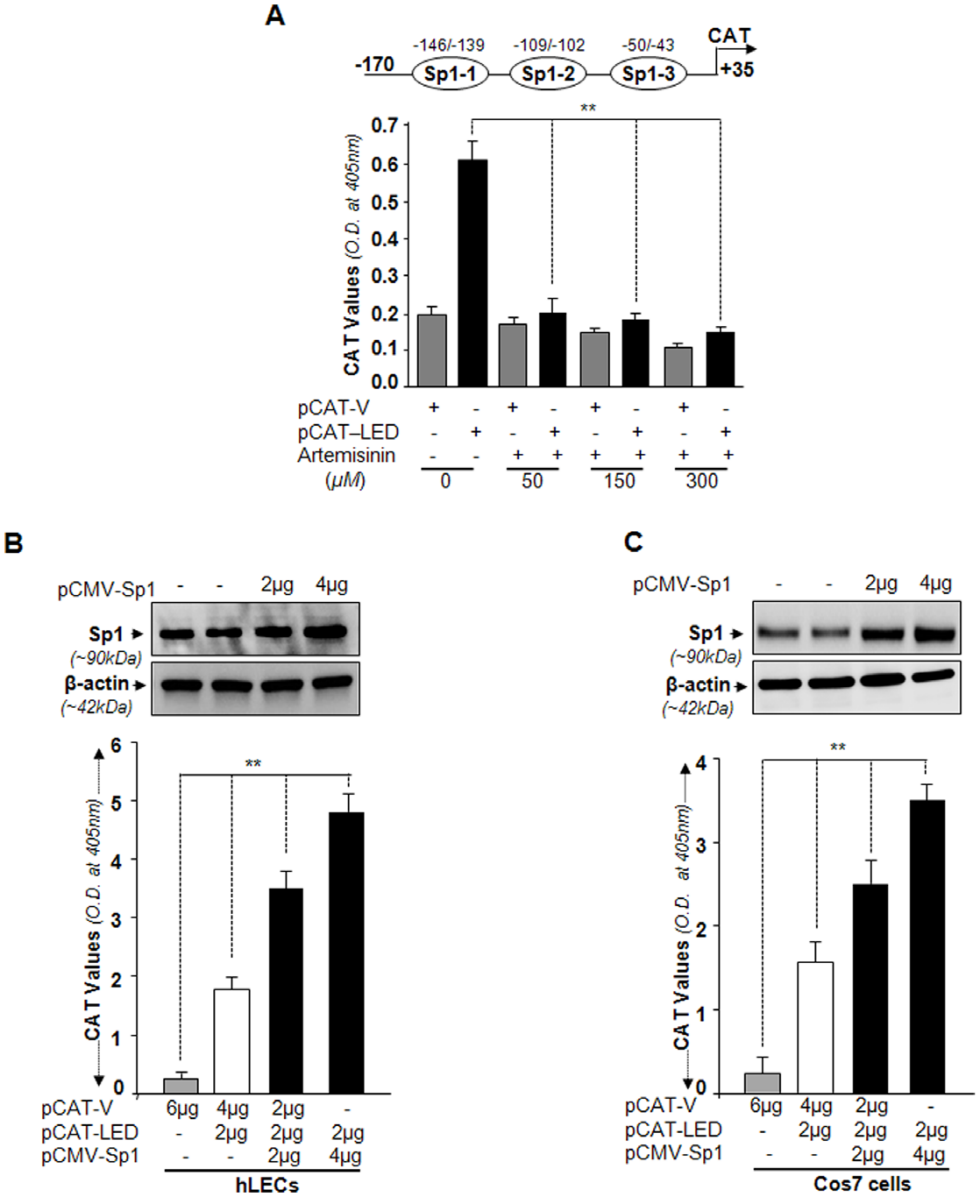
Sp1 expression levels had an impact on modulation of *LEDGF/p75* promoter activity. A, Interrupting Sp1 activity by artemisinin interrupted *LEDGF/p75* promoter activity in a concentration-dependent manner. *Upper* panel, a diagram of the *LEDGF/p75* promoter representing three Sp1-binding sites (−170/+35) used for CAT activity. A selective Sp1 inhibitor [33], artemisinin, reduced the activity of *LEDGF/p75* promoter in LECs in dose-dependent fashion. Artemisinin or its diluents (control) were added to culture medium of *LEDGF/p75* promoter constructs or empty vector transfected cells monolayer. Cells were disrupted and CAT activities were measured as described in the Materials and Methods section. Data are the mean of three experiments, and error bars indicate standard deviation (**p<0.001). B and C, Influence of Sp1 overexpression on transcriptional activity of *LEDGF/p75* promoter. Plasmid encoding pCAT-*LEDGF/p75* (−170/+35) or pCAT-V was cotransfected into hLECs (*B*) and Cos7 cells (*C*) with indicated amounts of pCMV-Sp1. Following CAT assay, CAT values were analyzed and represented as histograms, with Sp1 (black bar) or without Sp1 (open bar) overexpression. Empty CAT vector shows insignificant CAT activity (gray bar). Transfections were carried out as described in Materials and Methods and level of Sp1 protein was evaluated using Western analysis (B and C, Upper panel). The data are representative of at least three independent experiments. Each value represents the mean ± S.D. (**p<0.001).

Because ART inhibits gene transcription by attenuating/modifying Sp1 activity [33] and Sp1 selectively binds Sp1-response elements in the *LEDGF/p75* promoter (Figs. 4 and 5), we hypothesized that ART may abolish *LEDGF/p75* transcription, and thereby provide proof of the concept that Sp1 physically and functionally binds to *LEDGF/p75* promoter. To test this, we transfected LECs with *LEDGF/p75* gene promoter construct containing Sp1 response sites (−170/+35) fused to CAT reporter gene as described in the Materials and Methods section and reported earlier [20]. Cells were subjected to various concentrations of ART treatment (0, 50, 150, 300 µM) or to vehicle control. Analysis of CAT activity showed that ART strongly suppressed promoter activity of LEDGF/p75 even at the minimum concentration, 50 µM (Fig. 6A, black bar). This result is consistent with an earlier published report that ART blocks Sp1 transcriptional activity [33]. We concluded that loss of interaction of Sp1 with its *cis*-elements in the promoter may account for the loss of *LEDGF/p75* transcription as well as for downregulation of LEDGF/p75 protein (Fig. 1E).

We next examined the transcriptional effects of cellular abundance of Sp1 on the magnitude of *LEDGF/p75* transcription. Cells were cotransfected with *LEDGF/p75* promoter (−170/+35) containing Sp1 sites along with different concentrations of pCMV-Sp1. Cells cotransfected with Sp1 constructs caused robust induction of CAT activity, which increased with increased abundance of Sp1 (Fig. 6B upper panel, Western blot; lower panel, black bars). Next, we tested Sp1 activation of the same promoter in Cos7 cells (Fig. 6C upper panel, Western blot image; lower panel, black bars). Comparison of Fig. 6B and 6C reveals that the effect of Sp1 on *LEDGF/p75* promoter transcription was similar in both cell types, while cells not overexpressed with Sp1 showed basal levels of CAT activity (Fig. 6 B and C, open bar). These results demonstrated that Sp1 sites present in *LEDGF/p75* promoter were the functional Sp1 binding sites, and were responsive to cellular abundance of Sp1.

### Transcriptional analysis of *LEDGF/p75* gene promoter in Drosophila cell lines showed that Sp1 functionally determined *LEDGF/p75* transcription

**Figure 7:**
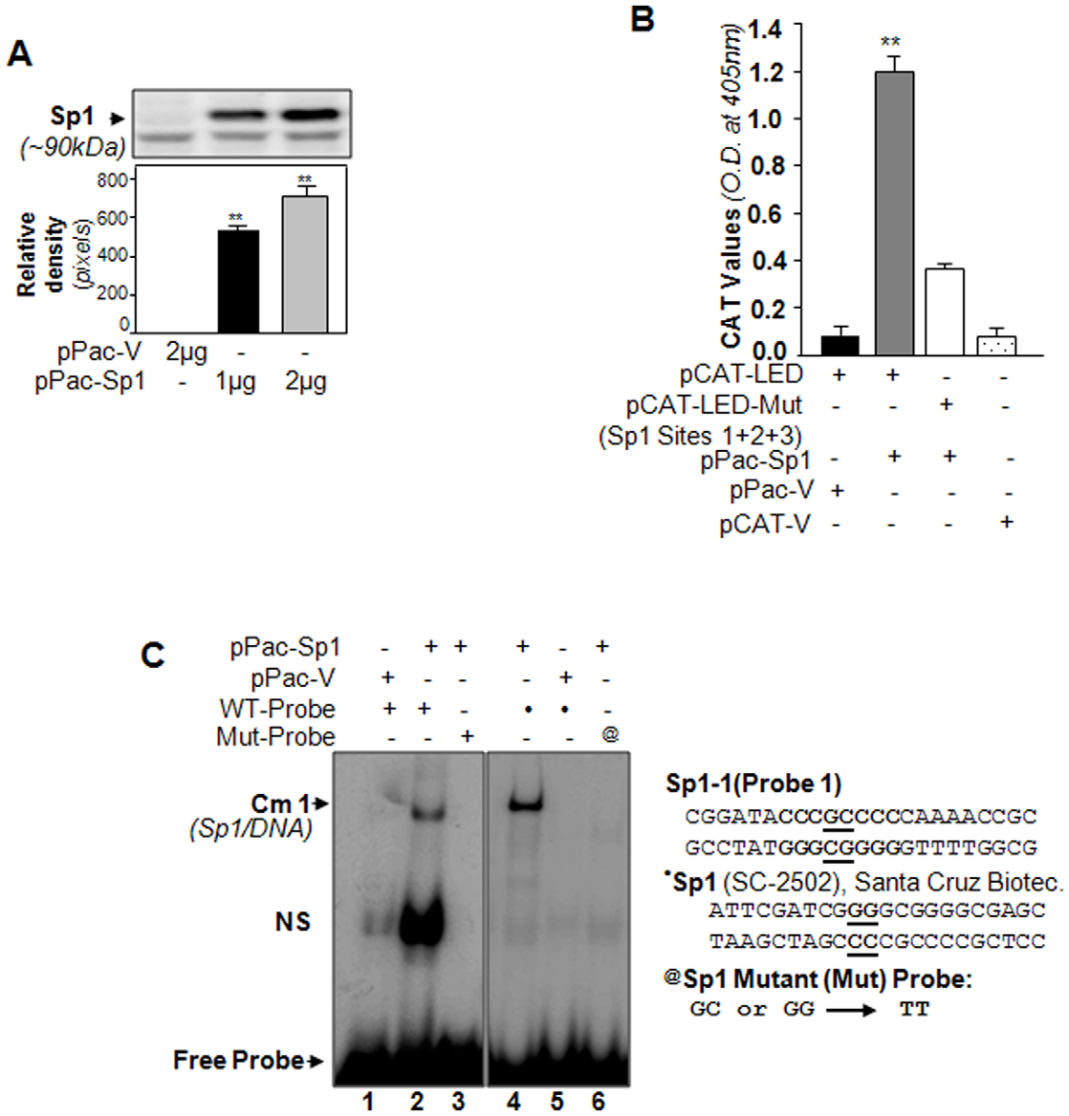
Sp1 expression in Sp1-deficient SL2 cells showed that Sp1 transactivated *LEDGF/p75* promoter by direct binding to its sites. A, SL2 cells, a *Drosophila* cell line, were transfected with indicated amounts of pPac-Sp1 (lanes 2 and 3) or pPac-V (lane 1). The expression level of Sp1 protein was examined by Western blot. Relative band density is shown below (A, gray bar *vs* black bar). B, Increasing Sp1 expression selectively increased *LEDGF/p75* promoter activity in SL2 cells. SL2 cells were cotransfected with pPac-Sp1 or pPac-vector (pPac-V) and pCAT-*LEDGF/p75* wild-type (pCAT-LED) or its mutant (pCAT-LED-Mut) reporter plasmid or pCAT vector (pCAT-V). Cells were processed to assay CAT activity as described in Materials and Methods. Results were expressed relative to activity of the LEDGF/p75 reporter activity in the presence of pPac-V or pPac-Sp1 and are presented as histograms: pPac-Sp1 with WT promoter (gray bar), and pPac-Sp1 with mutant promoter activity (open bar). The results are mean ± S.D. of three independent experiments (** *p<0.001*). C, Sp1 directly and exclusively bound to its sites in *LEDGF/p75* promoter. Nuclear extract was isolated and processed and then incubated with radio-labeled DNA probe containing Sp1 site (Probe 1 or standard control probe, ^•^Sp1). Nuclear extract from pPac-Sp1 overexpressed cells bound strongly to probe containing wild-type Sp1 consensus sequence (lane 2), but nuclear extract from pPac-vector transfected cells showed no binding with either wild-type or mutant probe (lanes 1 and 3). *Right* panel, Nuclear extracts of pPac-Sp1 transfected cells incubated with standard control probe containing Sp1 site (sc-2502, lane 4) or its mutant (sc-2503, lane 5) and nuclear extract from pPac vector transfected cells incubated with standard probe (lane 6).

Sp3 is a ubiquitous transcriptional protein that is highly homologous to Sp1 [24] and competes for the same DNA elements. To exclude the possibility of Sp3 involvement, we utilized Sp1-deficient *Drosophila* cell line (SL2), even though gel-mobility depletion assay showed no Sp3 involvement on identified Sp1 sites of *LEDGF/p75* promoter (Fig. 4B). To address the selective regulation of *LEDGF/p75* promoter by Sp1, we cotransfected SL2 cells with *LEDGF/p75* promoter linked to CAT reporter construct or its mutant in the presence or absence of expression plasmids encoding Sp1 (pPac-Sp1) or pPac-vector. Thus the transactivation of the *LEDGF/p75* promoter-containing Sp1 sites was largely dependent on the ectopically introduced transcriptional protein, Sp1. Analysis of data revealed that transactivity of *LEDGF/p75* promoter (−170/+35) was noticeably stimulated (∼12 fold) in pPac-Sp1 transfected cells (Fig. 7B, gray bar *vs* black bar), whereas residual activity remained with mutant construct (mutated at all sites, Sp1-Mut-1+2+3). This nonspecific activity may be related to certain unidentified factors in SL2 cells. However, CAT-basic vector was not activated by Sp1.

Additionally, we prepared cell extract from Sp1 transfected *Sp1-deficient* SL2 cells and conducted DNA-Sp1 binding assay. Cell extract isolated from SL2 cells ectopically expressing Sp1 was used after verification of Sp1 expression (Fig. 7A). Fig. 7C shows that ^32^p-radiolabeled probe containing Sp1 sites interacted with cell extract and formed complex (Cm 1) (Fig. 7C, lane 2), while nuclear extract from Sp1-deficient SL2 cells or mutant probe did not (Fig. 7C, lanes 1 and 3), demonstrating that Sp1 regulatory elements in the *LEDGF/p75* promoter are sites for Sp1 binding. Similar results were obtained with the other two Sp1 sites (Sp1-2 and Sp1-3, data not shown). As standard control, oligonucleotides containing the Sp1 sites (5′-ATTCGATCGU**GG**UGCGGGGCGAGC-3′; catalog number sc-2502; Santa Cruz Biotech) and its mutant ‘**GG**’ to ‘**TT**’ (sc-2503) were used to verify the results. Gel-shift assay demonstrated that nuclear extract isolated from SL2 cells ectopically expressing Sp1 was able to strongly bind to wild-type Sp1 probe, forming Cm1 complex (Fig. 7C, lane 4); nuclear extract from Sp1-deficient SL2 cells or mutant probe did not, indicating the integrity of the experiments. A band (NS) appeared signifying nonspecific entity or changes in nuclear proteins in SL2 cells following Sp1 transfection that nonspecifically gained the DNA-binding property. Taken together, data reveal that transcriptional protein Sp1 transactivated human *LEDGF/p75* promoter by directly binding to its sites.

### Sp1 Sumoylation negatively regulated *LEDGF/p75* gene transcription

**Figure 8:**
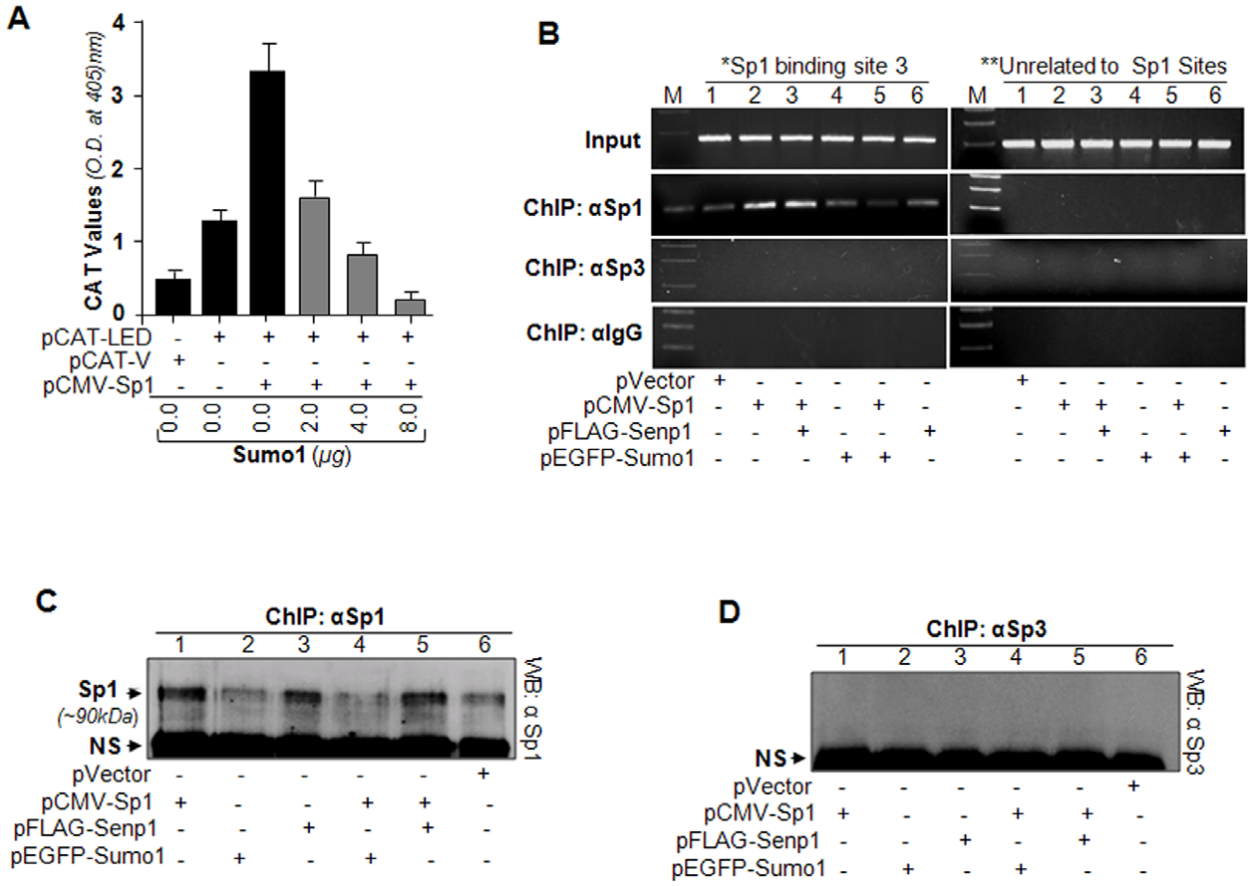
Sumoylation of Sp1 repressed *LEDGF/p75* gene transcription by decreasing the abundance of Sp1 to DNA. A, Repression of *LEDGF/p75* transcription by Sp1 Sumoylation. Cells were transfected or cotransfected with pCAT-*LEDGF/p75* (pCAT-LED) or pCAT vector (pCAT-V) and/or with increasing amounts of a plasmid encoding Sumo1 (pEGFP-Sumo1) as indicated. Cells were disrupted at predefined times and processed for CAT assay. Data indicate CAT activity in cells overexpressing different amounts of Sumo1 (A, gray bars) and without Sumo1 (black bars). Experiments were performed three times, and data are presented as mean ± S.D. B, ChIP assay coupled with desumoylation and DNA-protein complex dissociation experiments showed that the effect of Sumo1 on the abundance of Sp1 was concentration-dependent. hLECs were transfected with either pCMV-Sp1 alone or cotransfected with pEGFP-Sumo1 or pFLAG-Senp1. ChIP assay was performed in duplicates from each sample with anti-Sp1 or anti-Sp3 antibody or control IgG. Following processing, one set of precipitated samples was submitted for PCR analysis of Sp1 responsive region of *LEDGF/p75* promoter (B) as described in Materials and Methods. In another set of experiments, DNA bound proteins were eluted with high salt solution, and Western analysis was performed on elutes to measure Sp1 prevalence by anti-Sp1-antibody (C: lane 1, pCMV-Sp1; lane 2, pEGFP-Sumo1; lane 3, pFLAG-Senp1; lane 4, pCMV-Sp1 plus pEGFP-Sumo1; lane 5, pCMV-Sp1 plus pFLAG-Senp1; p-vector). Images shown in the panel (B) are of representatives of Sp1-3 (site 3) region. Similar results were obtained with Sp1-1 (site 1) and Sp1-2 (site 2) in the *LEDGF/p75* promoter when ChIP-PCR analysis was done (data not shown). Following stripping of Sp1 immunoblotted membrane, the same membrane was reprobed with Sp3 specific antibody, and no bands were observed (D).

Several recent studies have shown that Sumoylation of Sp1 represses gene transcription by regulating Sp1 expression [29], [38]. To study how this modification of Sp1 affects *LEDGF/p75* transcription, we cotransfected hLECs with *LEDGF/p75* promoter-CAT construct (−170/+35) and plasmid encoding pCMV-Sp1 and/or Sumo1 (pEFGP-Sumo1) in increasing amounts (2, 4, and 8 µg). Fig. 8 illustrates a decrease in *LEDGF/p75* promoter activity with increasing concentrations of Sumo1 (Fig. 8 A, gray bar). Results indicate that the CAT effect driven by *LEDGF/p75* promoter upon the expression of Sp1 was repressed by Sumo1.

To determine if Sumoylation/desumoylation affects the DNA-binding activity of Sp1 *in vivo*, we carried out a ChIP assay. Following transfection/cotransfection of hLECs with required encoding plasmids (pCMV-Sp1 and/or pEGFP-Sumo1 and/or pFLAG-Senp1 or their controls), we prepared chromatin samples as described in the Materials and Methods section. The Sp1 antibody, Sp3 antibody or control rabbit IgG immunoprecipitated complex were processed and analyzed by PCR using primers specific to promoter region. Fig. 8B shows that Sumo1 overexpression significantly reduced Sp1 binding with the endogenous *LEDGF/p75* gene (lanes 1 and 5), and this binding was increased in cells overexpressing Sp1 (lane 2) or Senp1 (lane 3). In contrast, the region not related to Sp1 did not provide detectable interaction between Sp1 and *LEDGF/p75* promoter. In another experiment, following immunoprecipitation with Sp1 or Sp3 antibody or IgG, the chromatin-bound proteins were eluted with high salt buffer, and were electrophoretically resolved and analyzed by immunoblot using Sp1 or Sp3 specific antibody. Analysis revealed that Sumo1 overexpression significantly reduced the level of Sp1, making it less available to *LEDGF/p75* gene promoter (Fig. 8C, lanes 2 and 4). In contrast, an abundance of Sp1 protein was detected in cells overexpressed with Sp1 or Senp1. These data were consistent with previous studies reporting that Sumoylation of Sp1 reduces the abundance of cellular Sp1, leading to negative regulation of Sp1 target gene transcription [29]. No band was obtained or detected when the membrane was reprobed with Sp3 antibody (Fig. 8D). Similar results were obtained with two other Sp1-2 and Sp1-1 sites in *LEDGF/p75* promoter (data not shown).

### RNA-interference and Sp1 overexpression experiments revealed that LEDGF/p75 was essential for hLEC survival in UVB-induced stress

**Figure 9:**
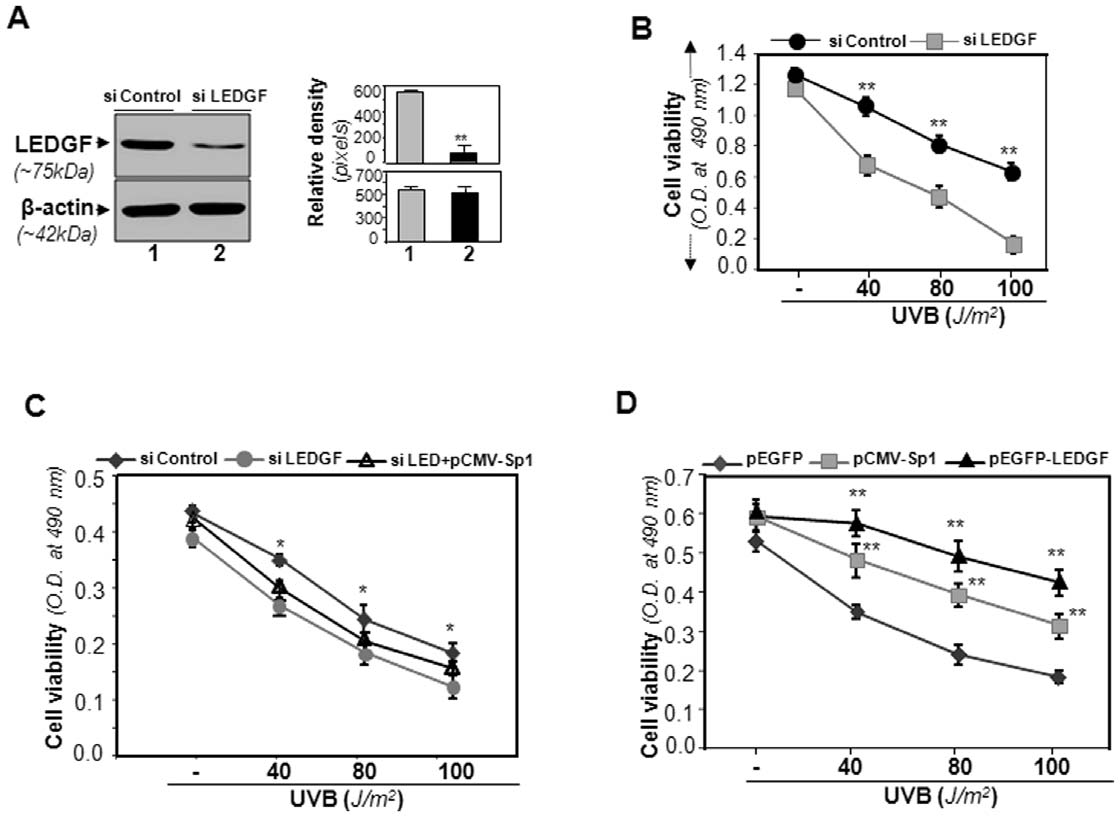
Small interfering RNA (siRNA) silencing of LEDGF/p75 revealed that hLECs were vulnerable to UVB-induced injury. A, hLECs were transfected with either mock, negative control siRNA or LEDGF/p75 siRNA. Following transfection, cell extracts were prepared and expression was examined by Western analysis using Anti-LEDGF/p75 antibody (A). Relative density in pixels is shown on the *right*. B, Control siRNA (siControl) or LEDGF/p75 siRNA (siLEDGF/p75) transfected cells were seeded in 12-well plates and submitted to UVB exposure as described in Materials and Methods. A survival assay-MTS assay was conducted, and data shown are mean ± S.D. values of three independent experiments. ** p<0.001 compared with control siRNA. C, Sp1 overexpression in cells with siRNA LEDGF/p75 conferred resistance against UVB stress. LEDGF/p75 siRNA transfected hLECs were transiently re-transfected with pCMV-Sp1 and then exposed to UVB stress. MTS assay was performed to evaluate vulnerability. * p<0.01 compared with respective controls. D, Sp1 or LEDGF/p75 overexpression in hLECs provided cytoprotection against stress induced by UVB. Cells were cultured and exposed to different doses of UV stress as indicated. Cell viability was analyzed using MTS assay as described in Materials and Methods. ** p<0.001 compared with respective controls. Data represent mean ± S.D. from three independent experiments.

LECs, the outermost cellular layer of the human lens, are exposed maximally to UV irradiation, suggesting that these cells are under continuous oxidative stress [39]. Studies have shown that LEDGF/p75's physiological expression level is vitally important, as it provides cytoprotection [5], [19], [40]. To study whether reduced expression of LEDGF/p75 influences cellular viability, we employed siRNA strategy to knock down the LEDGF/p75 expression in hLECs. The hLECs were transfected with the vector-based siRNA carrying selection marker [41]. Stably transfected cells were screened for LEDGF/p75 expression by Western analysis (Fig. 9A, *left* panel: images; *right* panel: histogram, densitometry of the band). The cells with reduced levels of LEDGF/p75 when exposed to UVB were less viable as revealed by MTS assay (Fig. 9B). Interestingly, overexpression of Sp1 to these cells failed to offer significant protection (Fig. 9C), while normal cells overexpressing LEDGF/p75 or Sp1 showed significant resistance against UV radiation-induced stresses compared to control, pEGFP empty vector transfected cells, indicating that cellular protection against UVB stress was caused by overexpression of LEDGF/p75 or Sp1-dependent increased expression. The results revealed that Sp1, a regulator of *LEDGF/p75* gene transcription, can be manipulated, potentially for development of Sp1-dependent LEDGF/p75 expression-based therapeutics.

## Discussion

LEDGF/p75 is a nuclear protein expressed in many cell types [2], [6], [7]. It enhances cellular survival by increasing the expression of stress-associated genes and small heat shock proteins (hsps) through interacting stress response (STRE) and heat shock protein gene response elements (HSE) in these genes [2], [5], [6], [19], acting as a transcription factor. Recent evidence revealed that DNA binding activity of LEDGF/p75 is not limited to STRE or HSE in the stress-associated genes, but LEDGF/p75 also binds to supercoiled DNA [22] and active chromatin markers as well as RNA polymerase II, and is associated with transcriptional activity of the transcriptional unit [21], [22]. *LEDGF/p75* gene inactivation has been shown to result in perinatal mortality and complex phenotype abnormalities [1]. LEDGF/p75's diverse and dynamic patterns of expression which account for its diverse mode of biological action are now well recognized. Less well understood are the processes by which expression levels of LEDGF/p75 are regulated. In the present study, we observed that LEDGF/p75 expression was modulated in hLECs isolated from eye lenses of different ages, and the expression pattern of LEDGF/p75 was well correlated with Sp1 expression levels (Fig. 1). Our studies of Sp1 overexpression and inhibition of Sp1 activity by artemisinin and Sp1 shRNA revealed that the modulation in expression of LEDGF/p75 protein or mRNA depended upon abundance of Sp1 expression (Fig. 1). These results argue that Sp1 can be a regulator of *LEDGF/p75* transcription. Sp1, a C2H2 zinc finger-containing factor, is a constitutively expressed protein that naturally engages in transregulating various TATA-less or TATA box-containing gene promoters. The promoter regions of these human genes are usually GC-rich, and, by definition, these genes are expressed ubiquitously [42], [43].

As expected, *in silico* analysis has identified three Sp1 sites within the GC-rich proximal promoter region of *LEDGF/p75* gene (−170/+1), and has revealed that *LEDGF/p75* is devoid of TATA-and CCAAT-boxes (Fig. 2), consistent with its constitutive expression in LECs and many other cell types [6], [20], [44]. A comprehensive and comparative analysis of sequences within 5′-flanking region of *LEDGF/p75* gene by DNA (ClustalW, a Web-based program for DNA sequence) revealed that the region with GC-Boxes and without TATA and CCAAT boxes is well conserved in mammals, further emphasizing the importance of Sp1 sites within the region (Fig. 2B). Based on our initial study (Fig. 1), coupled with bioinformatics analyses showing the presence of three putative Sp1 regulatory elements (Sp1-1, Sp1-2, Sp1-3) and Sp1 abundance-dependent increased expression of LEDGF/p75 mRNA (Fig. 1), we speculated that Sp1 sites may play a role in regulating LEDGF/p75. Deletion analysis showed that the promoter construct lacking Sp1 regulatory element (−28/+35) was not responsive and CAT activity was indistinguishable from CAT vector activity (Fig. 3A), indicating that *LEDGF/p75* transcription was dependent upon Sp1-DNA binding. More definitive evidence was produced by site-specific mutagenesis, which demonstrated that Sp1-1 (−146/−139) and Sp1-3 (−50/−43) have greater transactivation potential and enhance *LEDGF/p75* promoter activity. Surprisingly, however, Sp1-2 (−109/−102) acts as a repressor regulatory element, as disruption of its site enhanced promoter activity (Fig. 3B). Among all the Sp1 sites in *LEDGF/p75* promoter, Sp1-1 regulatory element showed the highest transactivation potential. However, overall, the promoter activity of LEDGF/p75 was increased, emphasizing that all three Sp1 regulatory elements are involved in controlling promoter activity. We believe that all three sites with their different transactivation potential are evolutionarily conserved for the purpose of controlling expression levels of LEDGF/p75. Additionally, we propose that the repressive regulatory element Sp1-2 is highly important for fine tuning of *LEDGF/p75* transcription. If this process goes awry, the result may be aberrant expression of LEDGF/p75.

Recently genome-wide analysis have indicated that the majority of genes contain multiple functional start sites, which are present in the core promoter region [35]. Furthermore, aberrant TSS usage has been associated with aberrant expression of transcripts and has been found to be linked to cellular abnormalities-such as cancer. In the current study, we found that LEDGF/p75 promoter consists of three TSSs: one major and two minor start sites (Fig. S1). We utilized major TSS sites to prepare deletion constructs of LEDGF/p75 promoter. However, another laboratory reported recently that LEDGF/p75 had different TSSs [45] from those that we found in lens epithelial cells (S1). Careful analysis revealed that TSS of LEDGF/p75 reported by previously published work by Desfarges et al [45] is located +208 nt downstream of TSS defined in our work (Fig. 2). We believe that the discrepancy may be due to the use of different cell types or cell background. Recently several genes have been found to have multiple transcription start points. A correlation between TATA-less promoters and multiple TSS usage has been generally accepted [46]. This feature of *LEDGF/p75* promoter demonstrates that its regulation is indeed complex. TSS may provide an extended transcriptional platform that can accommodate diverse transcriptional cofactors with different requirements of cells. However, transcription factor(s) and/cofactors may utilize distinct transcription initiation sites, and may be differentially regulated [47], in turn controlling the expression of genes such as LEDGF/p75.

Furthermore, more than 50% of human genes contain putative alternative promoters, and these genes are regulated by multiple promoters with different TSSs [48], [49]. Most of these genes belong to several cancer-associated genes such as MYC and BRCA1 [50], [51]. Several recent reports have indicated that LEDGF/p75 is associated with cancer, [3], [10], [40], [52], and its aberrant expression leads to cancer progression [52]. Based on our current work and that of [45], we can surmise that LEDGF/p75 contains alternative promoter (at least dual promoter) that influences the transcriptional level of LEDGF/p75 in the cell background. Interestingly, the promoter identified in our work has critically important features that are well conserved among mouse, rat and human cells: (i) it has a stretch of GC dinucleotide, (ii) it contains Sp1 responsive elements, and (iii) it does not contain TATA and CATAA boxes. Overall the structural characteristics of promoter strongly argue that it is evolutionarily conserved, and thus is functionally important and distinctly regulated. Comparison of the study presented here with recent reports [45] on regulation of *LEDGF/p75* gene transcription indicates that the *LEDGF/p75* gene may have alternative promoter that may function differently. However, further work is required to understand how and when *LEDGF/p75* promoter activity is altered in reference to cell background.

Our new TSS with alternative promoter is different from gene promoter recently reported [45], and redefining proximal promoter (Fig. 2). As defined in our current work, −170 bp of proximal promoter contained functional Sp1 responsive elements which greatly differ from those reported by Desfarges et al. [45]. In eukaryotes, alternative promoters have been documented for many tissue-specific and developmentally regulated genes in response to internal and external stimuli [53]. In most cases, transcripts originated from an active alternative promoter differ only in their 5′-untranslated region (UTR), but share the same coding sequence. Our new TSS adds as much as 208 bp to the 5′-UTR of LEDGF/p75 reported by Desfarges et al. [45]. This additional region of contains Sp1 sites responsible for *LEDGF/p75* transcription as reported by Desfarges et al. [45]. Moreover, an increasing number of studies identify the existence of alternate promoters for human genes and their differential usage as one important source of regulatory diversity [54]. We believe that the presence of multiple TSS or alternative promoter can add flexibility to the ways in which *LEDGF/p75* gene expression is regulated, can potentially affect translational efficiency, and can provide tissue, developmental, or signal specificity [53].

We found that the expression of LEDGF/p75 is transcriptionally tightly controlled by Sp1, and the regulatory elements of Sp1 that diverge from core consensus sequences contribute specifically to fine tuning the expression of LEDGF/p75. Analysis of *LEDGF/p75* promoter revealed that it contains nGGGCGGn or nCCA/GCCCn binding sites. Sp1 is known to recognize both elements with similar affinities [24]. DNA-protein binding assay using nuclear extracts from LECs demonstrated that Sp1 directly and specifically bound to Sp1 sites of *LEDGF/p75* promoter, and intensity of Sp1/DNA complex varied according to configuration of Sp1 regulatory element(s) (Fig. 4). This illustrates that binding affinity or intensity did influence activation potential of Sp1 elements, at least in regulating *LEDGF/p75* promoter. Based on the contribution of each Sp1 site in regulating *LEDGF/p75* transcription (Fig. 3B), we believe that each Sp1 site within *LEDGF/p75* promoter recruits different cofactors in context with cell background. Within the Sp family, Sp1 and Sp3 are ubiquitously expressed, and both can bind to the same cognate DNA-element [24], [36]. However, our *in vitro* DNA binding and ChIP assays excluded the possibility of Sp3 binding to either of the other Sp1 sites (Figs. 4 and 8). Our ChIP assay was not be able to dissect out Sp1 binding to each Sp1 site, since DNA fragments obtained in assay were limited to only 200 to 300 bps. However, we were able to define activity of all three sites of Sp1 using gel-shift and transactivation assays (Figs. 3, 4, 7). Furthermore, Sp1 has been shown to activate or repress target gene transcription, depending on the complex with which it interacts [24]. It is tempting to indicate that an analysis of each Sp1 binding site revealed that they are different from one another (but diverge from core binding sequences) and act differently in regulating *LEDGF/p75* transcription. We believe that the different activation potential of each site is due to recruitment of different factors or cofactors at the site (Figs. 2, 3, 4 and 5) [55]. However, data from Sp1-deficient SL2 cells that ectopically express Sp1, coupled with artemisinin (Fig. 7), indicates that Sp1 is indeed a regulator of *LEDGF/p75* transcription (Fig. 6A and B).

Furthermore, both the DNA binding and transactivation potential of Sp1 may be altered by its posttranslational modifications exemplified by phosphorylation, Sumoylation, glycosylation and acetylation [29], [38]. Our work showed that *LEDGF/p75* transcription is further controlled by Sp1 Sumoylation. Cellular or environmental stress may modify cellular signaling by mediating posttranslational modifications of proteins. Sumoylation of transcriptional protein has been shown to modulate transcriptional activity and affect gene expression and biological functions. In cotransfection and transactivation experiments, we found that Sumo1 reduced the transcriptional activity of Sp1 (Fig. 8A). This finding was supported by ChIP assay (DNA-Sp1 binding assay) using Sumo1 and Sumo hydrolase, Senp1, in which the DNA activity of Sp1 was increased significantly in cells transfected with Senp1. Additionally, in chromatin immunoprecipitation assay with anti-Sp1 antibody, we found that the reduced activity of *LEDGF/p75* transcription was caused by reduced interaction of Sp1 with *LEDGF/p75* promoter in cells overexpressing Sumo1 (Fig. 8B and C). These results are consistent with the known effect of Sp1 Sumoylation on transcriptional activity of other genes [29], [38].

To further study the functioning of LEDGF/p75 and its potential correlation with lens epithelial cell biology, we assessed the effect of LEDGF/p75 or Sp1 expression on survival of LECs facing stresses. By using LEDGF/p75 specific siRNA, we found that cells expressing reduced LEDGF/p75 level were more vulnerable to UVB-induced oxidative stress. Interestingly, stable transfection (siRNA-LEDGF/p75) of cells overexpressing pCMV-Sp1 also did not alter cell viability, while LECs overexpressed with either LEDGF/p75 or Sp1 showed significant resistance against UVB stress. These findings demonstrate that LEDGF/p75 plays a major role in protecting LECs from oxidative stress-induced cellular damage by UVB radiation (Fig. 9).

In summary, we have revealed, for the first time, that Sp1 specifically and differentially regulates human *LEDGF/p75* transcription, via directly binding its three *cis*-regulatory elements located in *LEDGF/p75* promoter. *LEDGF/p75* transcription is additionally controlled by regulatory activity of Sp1. The present characterization of *LEDGF/p75* gene and its interactions with Sp1 and the effect of Sp1 Sumoylation upon *LEDGF/p75* transcription is an initial step toward understanding the molecular mechanism governing the regulation of LEDGF/p75. Further detailed studies will be needed to delineate the mechanism involved in the expression of this physiologically important gene during different physiological conditions. Finally, the work presented here provides direct evidence that regulation of LEDGF/p75 is dependent upon Sp1 activity. Our data suggest that optimizing the expression level of LEDGF/p75 protein may be a useful therapeutic strategy in controlling cell abnormalities.

## Materials and Methods

### Cell Culture

Human lens epithelial cells (hLECs) (a gift of Dr. V. N. Reddy, Eye Research Institute, Oakland University, Rochester, MI) [56] and Cos7 cells (ATCC; CRL-1651) [57] were maintained routinely in our laboratory following the method described elsewhere [41]. Briefly, cells were cultured in a 75-mm tissue culture flask in Dulbecco's Modified Eagle's Medium (DMEM) supplemented with 15% heat-inactivated fetal bovine serum (FBS), 100 µg/ml streptomycin, and 100 µg/ml penicillin in a 5% CO_2_ environment at 37°C following standard methods. Cells were harvested and cultured in 96, 24, 48 or 6 well plates and 100 mm petri dishes according to the requirements of the experiment.

### Isolation of LECs from human subjects

Eye lenses were isolated from human subjects of variable ages ranging between 16 to 75 years, obtained from the Lions Eye Bank, Nebraska Medical Center. The lenses were divided into three age groups: group 1, 16 to 26 years (n = 4); group 2, 34 to 42 years (n = 3) and group 3, 52 to 75 years (n = 7). LECs from these subjects were generated as described earlier with some modification [58]. Briefly, clear lenses were washed with DMEM containing penicillin-streptomycin (100 µg/ml) and amphotericin B (25 µg/ml). Capsules were spread by forceps with cell layers upward on the surface of plastic culture petri dishes. Complete DMEM containing 15% fetal bovine serum was added. The growth of explants culture was monitored routinely. For subcultivating, monolayer of culture was incubated with trypsin (Gibco), and the dissociated cells were split as described earlier [59]. LECs obtained from 1 to 3 passages were used for the experiments.

This study, classified as *Research Involving Left-Over (Excess) Human Biological Material*, was approved by the ethics committee of the University of Nebraska Medical Center (Institutional Review Board [IRB] Approval ID: 247-09-NH). This study adhered to the tenets of the Declaration of Helsinki (2004).

*Drosophila* SL2 cells (CRL-1963) were purchased from American Type Culture Collection (Rockville, MD) and were maintained at room temperature in Schneider cell culture medium (Invitrogen, Carlsbad, CA) supplemented with 10% FBS and penicillin-streptomycin.

### Real-time PCR

Total RNA was isolated using the single-step guanidine thiocyanate/phenol/chloroform extraction method (Trizol Reagent; Invitrogen) and was converted to cDNA using Superscript II RNAase H^−^Reverse Transcriptase. Quantitative real-time PCR was performed with SYBR Green Master Mix (Roche Diagnostic Corporation, Indianapolis, IN) in a Roche® LC480 Sequence detector system (Roche Diagnostic Corporation). The comparative Cp method was used to calculate relative fold expression levels using Lightcycler ® 480 software, release 1.5.0 SP3. The Cps of target genes was normalized to β-actin as an endogenous control in each group. PCR conditions consisted of 10-min hot start at 95°C, followed by 45 cycles of 10 s at 95°C, 30 s at 60°C, and 10 s at 72°C. Primer sequence as follows: LEDGF/p75: Forward primer: 5′-CAGCAACAGCATCTGTTAATCTAAA-3′ and Reverse primer: 5′-GGGCTGTTTTACCATTTTGG-3′; Sp1: Forward primer: 5′-CCTGGATGAGGCACTTCTGT-3′ and Reverse primer: 5′-GCCTGGGCTTCAAGGATT-3′, β-actin: Forward primer: 5′-CCAACCGCGAGAAGATGA-3′ and Reverse primer: 5′-CCAGAGGCGTACAGGGATAG-3′.

### Expression constructs and transfections

Expression constructs, pCMV-Sp1, pPac-Sp1 and empty vectors (pCMV-V, pPac-V) were purchased from Addgene (Cambridge, USA). A construct containing a green fluorescent protein (GFP) and LEDGF/p75 cDNA was generated with the “living color system” (Clontech, Palo Alto, CA) using the plasmid vector pEGFP-C1 (Clontech). Similarly, full-length of Sumo1 cDNA was subcloned into pEGFP-C1 vector. The coding region of Sumo1 was amplified by PCR from human lens cDNA library using forward primer (5′-CCGTCGACATGTCTGACCAGGAG-3′) and reverse primer (5′-TCGGATCCGTTTTGAACACCACA-3′) with restriction enzyme sites, *Sal* I and *Bam* HI. The PCR product was digested and ligated into pEGFP vector. pFLAG-Senp1 was a generous gift from Dr. Yeh, University of Texas MD Anderson Cancer Center, Houston, TX. All transfection experiments were carried out either with Superfactamine Reagent (Qiagen) or Neon Transfection System (Invitrogen).

### Preparation of small interfering RNAs and transfection

The LEDGF/p75-specific small interfering (si)RNA expression plasmid was designed according to the method described earlier [41]. The sequence was selected from location 1340–1360 (5′-AAAGACAGCATGAGGAAGCGA-3′). The sense and antisense oligonucleotides with the internal loop were synthesized by Invitrogen. These were annealed and ligated into the *Bam H*I and *Hind*III sites of pSilencer 4.1-CMV hygro (Ambion). pSilencer 4.1- pCMVhygro expressing a scrambled siRNA (Ambion) was used as a control. One day after transfection, cells were subjected to a selection procedure using 400 µg hygromycine/ml over a period of 9 days with intermittent exposure. Knockdown of LEDGF/p75 was confirmed through Western analysis.

### Sp1 knockdown using Sp1 shRNA Plasmid (h)

Sp1 expression in human lens epithelial cells was silenced with Sp1 shRNA Plasmid (h) (Santa Cruz Biotechnology). Transfections were carried out with Neon transfection system (Invitrogen). Cell lysates were prepared 72 h after transfection, and silencing of Sp1 was confirmed through Western analysis. For selection of stably transfected cells, cells were treated with puromycin (5 µg/ml). After 3 days medium was replaced with freshly prepared selective media. 10 days later cells were washed with PBS (7.2) and cell lysate were submitted the western analysis and the association between levels of LEDGF/p75 and Sp1 protein expression was analyzed using Western analysis. shRNA (scrambled) was used as control.

### Preparation of LEDGF/p75 promoter-CAT construct

The genomic human phagemid P1 clone (Genomic System, St. Louis, MO) was used to construct 5′ flanking region of human *LEDGF/p75* gene as reported previously [20]. The genomic P1 clone comprising the *LEDGF/p75* gene was subjected to Genomic PCR with primers containing *Mlu* I and *Nhe* I, and a fragment encompassing −1239 and +35 bp was ligated to basic pCAT vector (Promega, Madison, WI) with the appropriate restriction enzymes as reported earlier [20]. Similarly, deletion constructs of different sizes (Fig. 3A, constructs: A, −170, B; −127, C; −63, D; −28 to +35) of *LEDGF/p75* promoter were prepared with appropriate sense primers bearing *Sac* I or *Mlu* I and antisense with *Nhe* I and ligated into pCAT-Basic vector. The plasmid was amplified using the standard method and was used for CAT assay.

To accurately locate the promoter region of human *LEDGF/p75* gene, we determined the transcription start site by the primer extension method. Briefly, the avian myeloblastosis virus (AMV) reverse transcriptase primer extension system (Promega) was used with poly (A)+RNA (50 µg) as template. RNA from hLECs was incubated with antisense oligonucleotide probe (5′-GTGGCTCCGAAGCGGATTTTCTGG-3′), and was end-labeled with T4 polynucleotide kinase (promega) and [Y-^32^p] ATP. The RNA was hybridized with Poly(A)+ RNA, and cDNA was synthesized following the company's protocol. Products were then heated for 10 min at 90°C in formamide loading buffer and analyzed on sequencing gel to determine the size and nucleotide base(s) of TSS. The fmol DNA Cycle Sequencing System (Promega) was used for sequencing reactions with the primers to read the position of extended product, and TSS was determined.

### Site-directed Mutagenesis (SDM)

PCR-based site-directed mutagenesis was performed using the QuickchangeTM Site-directed Mutagenesis kit (Stratagene, La Jolla, CA) following the company's protocol. We made mutations in Sp1 binding sites (Mut-1: GC changed to TT or CG changed to AA; Mut-2: CC changed to TA or GG changed to TA; Mut- 3: GC changed to TT or CG changed to AA). Briefly, the double-stranded *LEDGF/p75* promoter construct (−170/+35) was used as template DNA with a pair of complementary primers used to mutate the *LEDGF/p75* promoter construct with PCR. The primers used for mutation were as follows: Sp1-Mut-1_for′_5′-GAGGCCCGGATACCCGU**TT**UCCCAAAACCGCGTCCAC-3′; Sp1-Mut-1_rev′_5′-GTGGACGCGGTTTTGGGU**AA**UCGGGTATCCGGGCCTC-3′; Sp1-Mut-2_for′_5′-CAACAATCATTTCCTCCCTCU**TA**UCGCCTTTTACATACAGTAC-3′; Sp1-Mut-2_rev′_5′-GTACTGTATGTAAAAGGCGU**TA**UGAGGGAGGAAATGATTGTTG-3′; Sp1-Mut-3_for′_5′-GAGAAGGCCAGCAGGU**TT**UCGGGCCGGGCCCG-3′; Sp1-Mut-3_rev′_5′-CGGGCCCGGCCCGU**AA**UCCTGCTGGCTTCTC-3′.

### Transfection and Chloramphenicol Acetyltransferase Assay (CAT Assay)

CAT assay was carried out using a CAT-ELISA (Roche Diagnostics) kit. hLECs and Cos7 cells were cultured at a density of 5×10^5^ cells in 5 ml of DMEM containing 15% FBS per 60-mm petri dish in a 37°C incubator containing 5% CO_2_. Cells were washed with the same medium and transfected/cotransfected with Superfactamine Reagent (Invitrogen) with promoter/CAT reporter constructs and/or GFP expression vector or pCMV-Sp1 or SiRNA construct specific to LEDGF/p75 or Sp1 along with 1 µg of pSEAP vector [57]. After 72 h of incubation, cells were harvested, and extract was prepared and protein concentration was normalized. CAT-ELISA was performed to monitor CAT activity. The absorbance was measured at 405 nm using a micro titer plate ELISA reader. The concentration of Plasmid DNA was equal in each transfection to maintain the similar DNA burden on cells and to avoid any nonspecific effect(s). Transactivation activities were adjusted for transfection efficiencies using SEAP values (OD; ex/em, 360/449).

### Preparation of Lens Epithelial Cell Nuclear Extract

Human LEC nuclear extract was prepared as described earlier [5], [20]. Briefly, human LECs (1×10^6^) were cultured in 100-mm plates. The cells were washed gently with chilled phosphate-buffered saline (pH 7.2). Cells were collected by centrifugation using a microcentrifuge and resuspended in 5 pellet volumes of cytoplasmic extract buffer (10 mM HEPES, 60 mM KCl, 1 mM EDTA, 0.075% (v/v) Nonidet P-40, 1 mM phenylmethylsulfonyl fluoride, adjusted to pH 7.6). After a short incubation on ice, the cytoplasmic extract was removed from the pellet. Following washing with cytoplasmic extract without detergent (Nonidet P-40), the fragile nuclei were resuspended in nuclear extract buffer (20 mM Tris-HCl, 420 mM NaCl, 1.5 mM MgCl_2_, 0.2 mm EDTA, 1 mM phenylmethylsulfonyl fluoride, and 25% (v/v) glycerol, adjusted to pH 8.0). Salt concentration was adjusted to 400 mM using 5 M NaCl, and the extract was incubated on ice for 10 min with occasional vortexing. Finally, the extract was spun at 14,000 rpm for 30 min to pellet the nuclei. Protein was estimated according to the Bradford method, and extract was used for EMSA.

### Western blot and antibodies

Total cell lysates were prepared in ice-cold radio immunoprecipitation assay (RIPA) lysis buffer, as described previously [19]. Equal amounts of protein samples were resolved onto a 10% SDS gel, blotted onto PVDF membrane (Perkin Elmer, Waltham, MA), and immunostained with primary antibodies at the appropriate dilutions of LEDGF/p75 monoclonal antibody (BD Biosciences, USA), and Sp1 monoclonal antibody (Santa Cruz Biotechnology). Membranes were incubated with horseradish peroxidase-conjugated secondary antibodies (1∶1500). Specific protein bands were visualized by incubating the membrane with luminol reagent (Santa Cruz Biotechnology) and recorded with FUJIFILM-LAS-4000 luminescent image analyzer (FUJIFILM Medical System Inc, USA). To ascertain comparative expression and equal loading of the protein samples, the membrane stained earlier was stripped and reprobed with β-actin antibody (Abcam, USA).

### Electrophoretic Mobility Shift Assay (EMSA)

To perform gel-shift assay, oligonucleotides containing Sp1 binding elements and respective mutant probes were synthesized commercially, annealed, and end-labeled with [γ-^32^P] ATP using T4 polynucleotide kinase (New England Biolabs, Inc.). The binding reaction was performed in 20 µl of binding buffer containing 20 mM Tris-HCl (pH 8.0), 75 mM KCl, 5% glycerol, 50 µg/ml bovine serum albumin, 0.025% Nonidet P-40, 1 mM EDTA, 5 mM DTT, and 1 µg of poly (dI/dC). Five fmol (1000 cpm) of the end-labeled probe were incubated on ice for 30 min with 10–1000 ng of the GST-LEDGF/p75 fusion protein. Samples were then loaded on 5% polyacrylamide gel in 0.5× TBE buffer for 2 h at 10 V/cm. The gel was dried and autoradiographed.

### Chromatin Immunoprecipitation (ChIP) Analysis

ChIP analysis was conducted by the ChIP-IT express kit (Active Motif). Cells were processed following the company's protocol. The fixation reactions were stopped by adding Glycine Fix-Stop solution. After washing with ice-cold PBS, cells were collected in solution containing PMSF, and centrifuged at 4°C. Cell pellet was disrupted with a Dounce homogenizer (10 strokes of 10 s each to aid in nuclei release) in 1 ml ice-cold lysis buffer containing protease inhibitor and PMSF. After centrifugation, nuclei were resuspended in shearing buffer (Active Motif) and incubated on ice for 10 min. Chromatin was then sheared to 200–300 bp using a closed system ultrasonic cell disruptor (Microson, Farmingdale, NY). Samples were centrifuged at 15,000 rpm for 10 min at 4°C, and the supernatant was stored at −80°C. An aliquot of this material was retained as “input” DNA. The remaining chromatin sample was divided; one-half was immunoprecipitated with the test antibody (Sp1+ ChIP grade antibody, Millipore), and the second half was used for a mock immunoprecipitation with a control IgG. ChIP assay bands were compared with assay bands obtained with the input DNA. Mock immunoprecipitation reactions were performed using control IgG (ChIP-IT Control Kit, Active Motif). Regions of the human *LEDGF/p75* promoter that contained Sp1 binding sites were amplified (Go-Taq, Promega) using specific primers. For comparison, a 222-bp sequence from the human *LEDGF/p75* promoter beyond 2 kb Sp1 binding site was also amplified from the IP and mock IP samples. Sequences of primers used in this experiment are shown in Fig. 5. ChIP assays were conducted via standard PCR amplification (Go-Taq) and agarose gel electrophoresis. Amplified DNA bands were resolved on 2.5% agarose gels, and images were obtained using FUJIFILM-LAS-4000 luminescent image analyzer (FUJIFILM Medical system Inc, USA). PCR band sizes were verified using a low molecular mass DNA ladder (Fermentas).

### Cell survival assay (MTS assay)

A colorimetric MTS assay (Promega, Madison, MI, USA) was performed as described earlier. This assay of cellular proliferation uses 3-(4, 5-dimethylthiazol-2-yl)-5-(3-carboxymethoxyphenyl)-2 to 4-sulfophenyl)-2H-tetrazolium salt. Upon being added to medium containing viable cells, MTS is reduced to a water-soluble formazan salt. The O.D. 490 nm values were measured after 4 h with an ELISA reader.

### Statistical method

Data are presented as Mean ± S.D. of the indicated number of experiments. Data were analyzed by Student's t-test. A *p* value of <0.01 and <0.001 was defined as indicating a statistically significant difference.

## Supporting Information

Figure S1 **Determination of the transcription start site of the LEDGF/p75 gene by primer extension.** Transcription start site was determined with primer extension analysis using commercial kit (Promega). The 5′-radiolabelled antisense nucleotide 5′-GAGGCACCGAAGCGGATTTTCTGG-3′ complementary to human LEDGF/p75 cDNA sequence was used as primer in a reverse transcription reaction with control *E. coli* tRNA (lane 1), and poly (A)+ RNA (lane 2) isolated from human LECs. Products obtained were resolved on 8% denaturating sequencing gel and subjected to autoradiography. M (lane 10): molecular weight markers. The arrow corresponds to the band at position 125(nt) size is the major transcription start site (TSS), and two minor TSS (90 and 76). Lanes 3, 4, 5 and 6 correspond to G, A, C, and T sequences respectively used to determine start sites.
(TIF)
