# Supplementary figures and images for: Transcriptional Protein Sp1 Regulates LEDGF Transcription by Directly Interacting with Its Cis-Elements in GC-Rich Region of TATA-Less Gene Promoter
Source: PLoS One. 2012 May 16;7(5):e37012. doi: 10.1371/journal.pone.0037012 (PMC3353957; doi:10.1371/journal.pone.0037012)

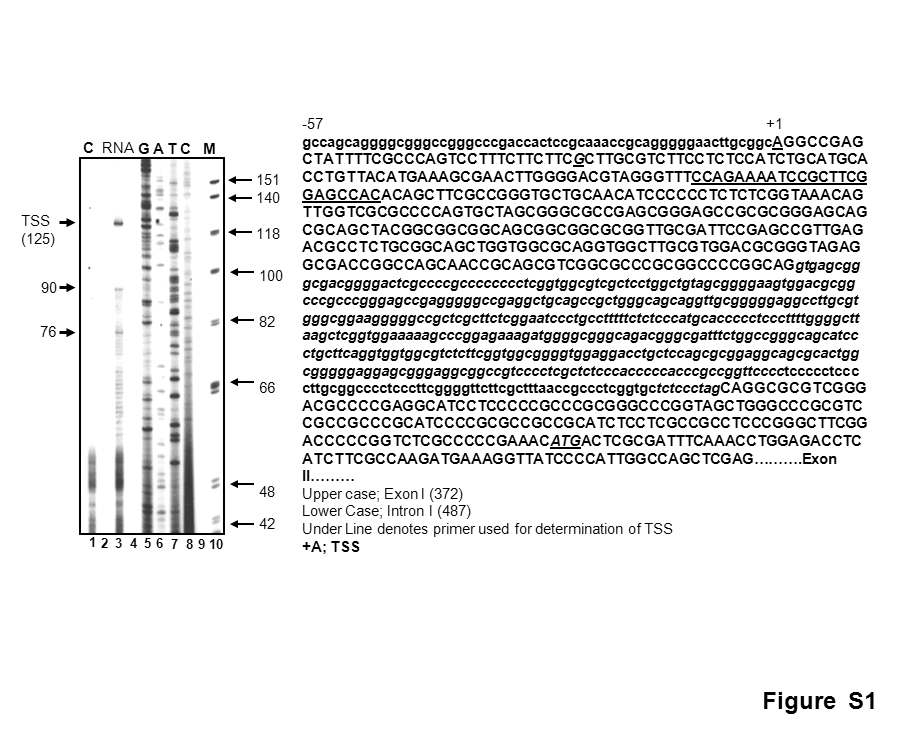

Supplement: Figure S1 — Determination of the transcription start site of the LEDGF/p75 gene by primer extension. Transcription start site was determined with primer extension analysis using commercial kit (Promega). The 5′-radiolabelled antisense nucleotide 5′-GAGGCACCGAAGCGGATTTTCTGG-3′ complementary to human LEDGF/p75 cDNA sequence was used as primer in a reverse transcription reaction with control E. coli tRNA (lane 1), and poly (A)+ RNA (lane 2) isolated from human LECs. Products obtained were resolved on 8% denaturating sequencing gel and subjected to autoradiography. M (lane 10): molecular weight markers. The arrow corresponds to the band at position 125(nt) size is the major transcription start site (TSS), and two minor TSS (90 and 76). Lanes 3, 4, 5 and 6 correspond to G, A, C, and T sequences respectively used to determine start sites. (TIF) [file pone.0037012.s001.tif]
